# Targeting innate immunity in cancer: mechanistic foundations, therapeutic vaccination, and clinical translation

**DOI:** 10.3389/fimmu.2026.1941235

**Published:** 2026-09-18

**Authors:** Xinghan Li, Yitong Meng, Jijun Dong, Yue Ma, Dequan Liu, Xiaodong Zhang, Lingjie Zhao

**Affiliations:** 1 Department of Stomatology, General Hospital of Northern Theater Command, Shenyang, China; 2 Department of Urology, The First Affiliated Hospital of Dalian Medical University, Dalian, China

**Keywords:** cancer immunotherapy, cancer vaccines, innate immune sensing, innate immunity, tumor microenvironment

## Abstract

Cancer evolves within tissue ecosystems in which innate immune surveillance and tumor-promoting inflammation jointly shape disease progression and therapeutic response. Innate receptors detect cellular stress, altered-self ligands, damaged-cell products and tumor-derived nucleic acids, thereby initiating cytotoxicity, phagocytosis, antigen presentation and adaptive immune priming. However, tumors can redirect these mechanisms through suppressive cytokines, metabolic competition, hypoxia and dysregulated chemokine networks, promoting immune exclusion, metastasis and treatment resistance. This Review examines innate recognition, immunoediting and innate–adaptive crosstalk. We discuss the heterogeneity, dysfunction and therapeutic targeting of dendritic cells, macrophages, natural killer cells, neutrophils, myeloid-derived suppressor cells, innate lymphoid cells and other tissue-resident populations. We also evaluate Toll-like receptor, cGAS–STING, RIG-I–MAVS, inflammasome and complement pathways as therapeutically actionable but potentially tumor-promoting signaling systems. Finally, we consider how innate adjuvants, antigen selection, delivery technologies and rational combinations can improve therapeutic cancer vaccines. Emerging clinical studies demonstrate feasibility, but inconsistent efficacy and treatment-associated toxicity emphasize the need for biomarker-guided patient selection, spatially restricted delivery and mechanism-based sequencing. Clinical translation will depend on biomarker-guided patient selection, localized delivery and treatment sequencing that preserves antimicrobial defense and tissue homeostasis.

## Graphical Abstract

**Figure:**
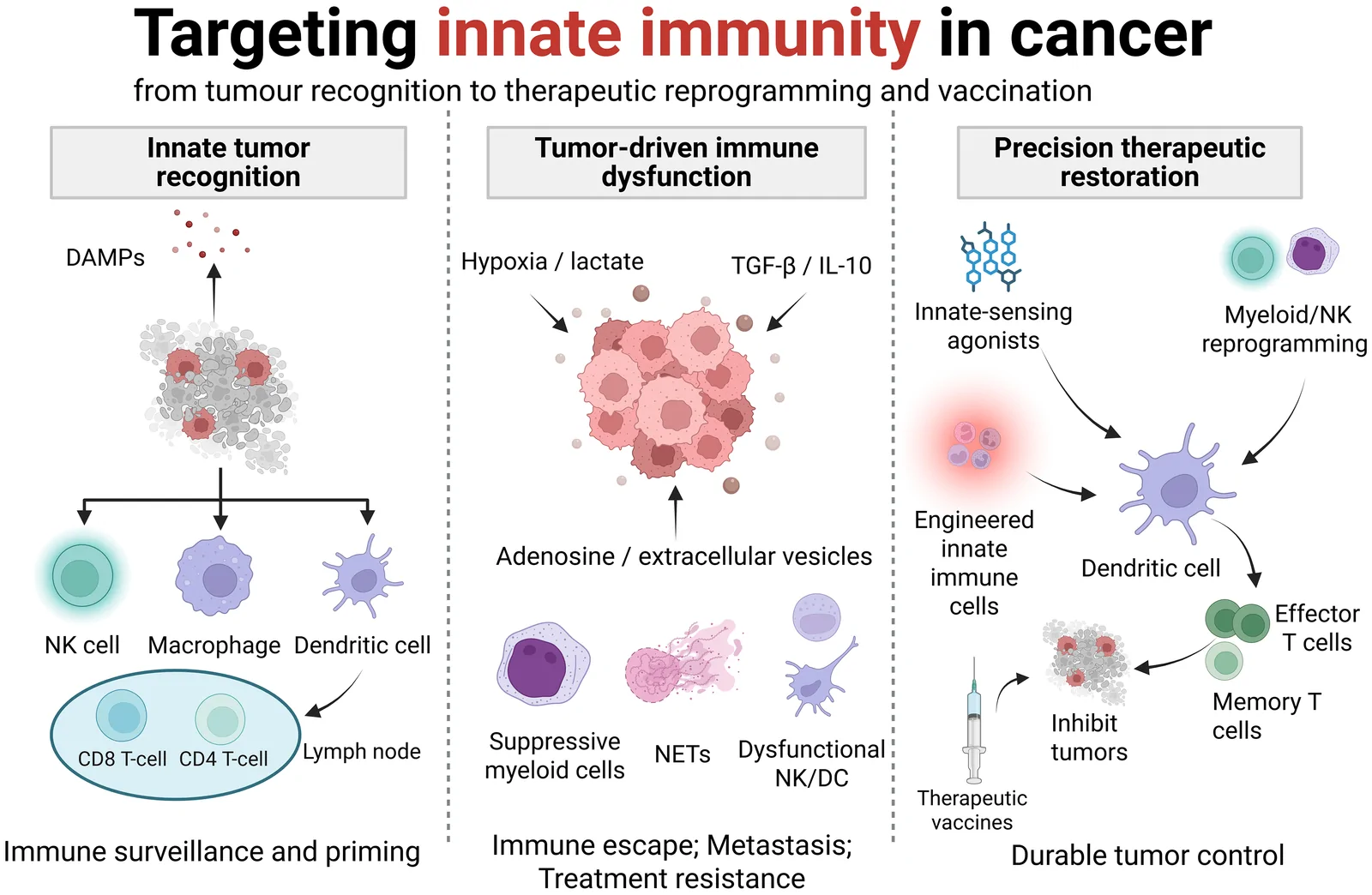
Targeting innate immunity in cancer: from tumor recognition to therapeutic reprogramming and vaccination. Innate immunity exerts context-dependent effects throughout cancer development and treatment. Transformation-associated stress and tumor-derived DAMPs activate NK-cell cytotoxicity, macrophage phagocytosis and DC antigen uptake and presentation, thereby supporting immune surveillance and CD4^+^- and CD8^+^-T-cell priming. As tumors progress, hypoxia, lactate accumulation, TGF-β, IL-10, adenosine and extracellular vesicles remodel the innate immune compartment. The resulting suppressive myeloid populations, NET-forming neutrophils and dysfunctional NK cells and DCs promote immune escape, metastatic dissemination and treatment resistance. Precision therapeutic strategies aim to reverse these processes through innate-sensing agonists, myeloid- and NK-cell reprogramming, engineered innate immune cells and therapeutic cancer vaccines. These interventions converge on DC activation, effector-T-cell recruitment and memory formation, ultimately promoting tumor regression and durable immune control. DAMP, damage-associated molecular pattern; DC, dendritic cell; IL, interleukin; NET, neutrophil extracellular trap; NK, natural killer; TGF-β, transforming growth factor-β. Created in BioRender. Liu, D. (2026) https://BioRender.com/wlf1dlh.

## 1 Introduction

The clinical success of immune-checkpoint blockade has established immune evasion as a therapeutically actionable hallmark of cancer, while also exposing a fundamental limitation of T-cell-centred strategies: adaptive immunity can operate effectively only when malignant cells are first detected, tumor antigens are productively presented and effector lymphocytes are recruited into permissive tissue niches (1). These upstream processes are governed largely by innate immunity, which integrates altered-self ligands, damage-associated signals and perturbed nucleic-acid homeostasis through cytotoxic lymphocytes, phagocytes, dendritic cells (DCs) and germline-encoded pattern-recognition pathways. Their functions are neither lineage-fixed nor uniformly antitumor; instead, ontogeny, anatomical localization, metabolic state and signal duration determine whether innate populations eliminate transformed cells or promote fibrosis, immune exclusion and metastatic progression. Spatial profiling studies conducted in 2021 identified organized myeloid and lymphoid immune hubs in human colorectal cancer, whereas a 2025 pan-cancer analysis revealed recurrent profibrotic fibroblast–macrophage ecotypes associated with altered tumor immunity (2, 3). These multicellular niches directly influence therapeutic competence: analysis of hepatocellular carcinomas (HCCs) exposed to neoadjuvant PD-1 blockade linked treatment response to intratumoral DC–CD4^+^ T-cell niches that supported local CD8^+^ T-cell differentiation (4). Conversely, the identification of tumor-cell IGSF8 as an inhibitory ligand for NK-cell receptors demonstrated that malignant cells can acquire dedicated innate checkpoints that complement defects in antigen presentation and adaptive immune recognition (5).

Together, these findings identify intervention points in tumor sensing, antigen presentation,tissue remodeling and innate–adaptive crosstalk. Early clinical studies provide proof ofprinciple across distinct modalities: intravesical administration of the IL-15 superagonistnogapendekin alfa inbakicept with Bacillus Calmette–Guérin (BCG) produced durableresponses in patients with BCG-unresponsive non-muscle-invasive bladder cancer, whereas intratumoraladministration of the oncolytic herpesvirus G47Δ generated a survival signal in residual orrecurrent glioblastoma (6, 7). Platform development is simultaneously blurring the boundaries between innateactivation, immunogenic tumor destruction and vaccination. An engineered oncolytic virus carryingtumor-irrelevant memory-T-cell epitopes recruited bystander lymphocytes, promoted antigen spreadingand enhanced immunotherapy in preclinical models, while recent work showed that mRNA vaccinationcould induce type-I-interferon-dependent activation of antigen-presenting cells (APCs) and sensitizetumors to checkpoint blockade in mice; the accompanying retrospective clinical associations requireprospective validation (8, 9). Nevertheless, the same inflammatory programs may become suppressive when they are chronic, systemic or activated in inappropriate cellular compartments. This Review therefore integrates the mechanisms of innate tumor recognition and immunoediting with the spatial and functional plasticity of DCs, macrophages, NK cells, neutrophils, myeloid-derived suppressor cells (MDSCs) and innate-like lymphocytes. It further evaluates innate-sensing pathways, engineered-cell approaches and therapeutic cancer vaccines, arguing that durable clinical benefit will require cell-resolved biomarkers, compartment-selective delivery and treatment sequences aligned with antigen release, DC priming and adaptive effector recruitment (**Graphical abstract**).

## 2 Innate immune recognition, surveillance and adaptive crosstalk in cancer

Innate tumor immunity integrates direct recognition of malignant cells with indirect sensing of tumor-derived danger signals, antigen handling and early innate–adaptive crosstalk. Direct recognition begins with contact-dependent recognition of the malignant-cell surface. NK cells integrate transformation-associated activating ligands and diminished inhibitory HLA class I signaling, whereas macrophages interpret antibody-opsonization and CD47–SIRPα “don’t-eat-me” signaling to determine phagocytosis. Experimentally, STING activation enabled NK-cell rejection of MHC-I-deficient, CD8^+^ T-cell-resistant tumors, and a phase Ib study of CD47 blockade plus rituximab provided clinical proof of concept for macrophage-mediated phagocytosis in lymphoma (10, 11). By contrast, indirect sensing is initiated when endogenous danger signals—including cytosolic or extracellular DNA, cGAMP, ATP and other products of stressed or dying tumor cells—or microbial pathogen-associated molecular patterns activate cGAS–STING, Toll-like receptor, inflammasome or related pattern-recognition pathways in malignant, stromal or immune cells; tumor-derived cGAMP, for example, was shown to activate STING and IFN-β production in non-malignant myeloid and B cells, whereas intravesical BCG induced durable heterologous TNF and IL-1β responses in circulating cells from patients with non-muscle-invasive bladder cancer (12, 13).

Thus, direct recognition requires an effector–target synapse and immediately initiates cytotoxicity or phagocytosis, whereas indirect sensing can originate in bystander cells and amplify immunity through type I interferons, IL-15 and inflammatory chemokines; these pathways nevertheless converge because danger-sensing cells activate the NK cells and phagocytes that execute tumor clearance (11, 12). During the elimination phase of immunosurveillance, NK-cell cytotoxicity and macrophage phagocytosis remove susceptible transformed cells, particularly cells that have lost MHC-I-mediated protection or display accessible antibody-coated antigens, before a clinically detectable tumor is established (10, 11). Elimination is also selective. DNA barcoding of murine mammary cancers demonstrated that endogenous immunity and checkpoint therapy markedly restricted clonal diversity and reproducibly enriched pre-existing resistant clones with distinct immune-evasion programs during primary growth and metastasis (14). These findings support a dynamic interpretation of immune equilibrium in which continuing immune pressure restrains tumor burden while selecting less-visible clones, until tumor-intrinsic escape mechanisms combine with local effector dysfunction—illustrated by the rapid loss of cytotoxicity, IFN-γ and CCL5 after NK cells enter established tumors—to permit progressive disease (14, 15).

Innate–adaptive crosstalk is organized principally around DC handling of tumor antigens. cDC1 cells acquire cell-associated malignant material. They cross-present processed peptides through MHC-I to CD8^+^ T cells and present associated antigens through MHC-II to CD4^+^ T cells. CD4^+^-cell-derived CD40 licensing sustains the expansion and persistence of both tumor-specific populations (16). Antigen presentation continues within the tumor, where a CCR7-negative, CXCL9-producing cDC1 state forms stromal niches with TCF1^+^ stem-like CD8^+^ T cells, cross-presents tumor antigens and drives local effector differentiation; analogous cDC1–CD8^+^ clusters in human cancers were associated with checkpoint-blockade responsiveness and improved survival (17). Chemokine circuits determine whether this adaptive response can be established. Activated NK cells release XCL1 and CCL5 and provide FLT3L to recruit and sustain cDC1, tumor-derived prostaglandin E_2_ disrupts this NK–cDC1 axis, and cDC1-derived CXCL9–CXCR3 signals subsequently recruit and spatially organize tumor-reactive CD8^+^ cells (17, 18). Other innate–adaptive relationships are strongly subset- and context-dependent. Perivascular FOLR2^+^ tissue-resident macrophages colocalized with and activated CD8^+^ T cells in human breast cancer and were associated with favourable survival, whereas activated tumor-specific CD4^+^ T cells mobilized antitumor neutrophils that eliminated antigen-loss variants after OX40 costimulation or CTLA-4 blockade in melanoma models (19, 20). Finally, innate immunity can retain altered responsiveness without conventional antigen-receptor memory: intravesical BCG produced sustained cytokine, transcriptional and H3K4me3-associated reprogramming consistent with trained immunity in patients with bladder cancer, while donor-derived IL-12/IL-15/IL-18-conditioned memory-like NK cells remained detectable and functional for at least two months after transfer into patients with leukemia; these clinically relevant states should nevertheless be distinguished from classical antigen-specific T- and B-cell memory, and a causal relationship between trained-immunity magnitude and cancer outcome remains to be established (13, 21).

Although trained immunity provides a mechanistic framework for persistent innate reprogramming, its contribution to clinical tumor control remains uncertain. Prospective studies incorporating pathway-specific pharmacological or genetic perturbations are needed to determine whether trained-immunity programs contribute causally to tumor control rather than merely accompanying a broader inflammatory state.

The coordinated mechanisms linking malignant-cell recognition, innate effector activity, DC-dependent priming and cancer immunoediting are summarized in **Figure 1**.

**Figure 1:**
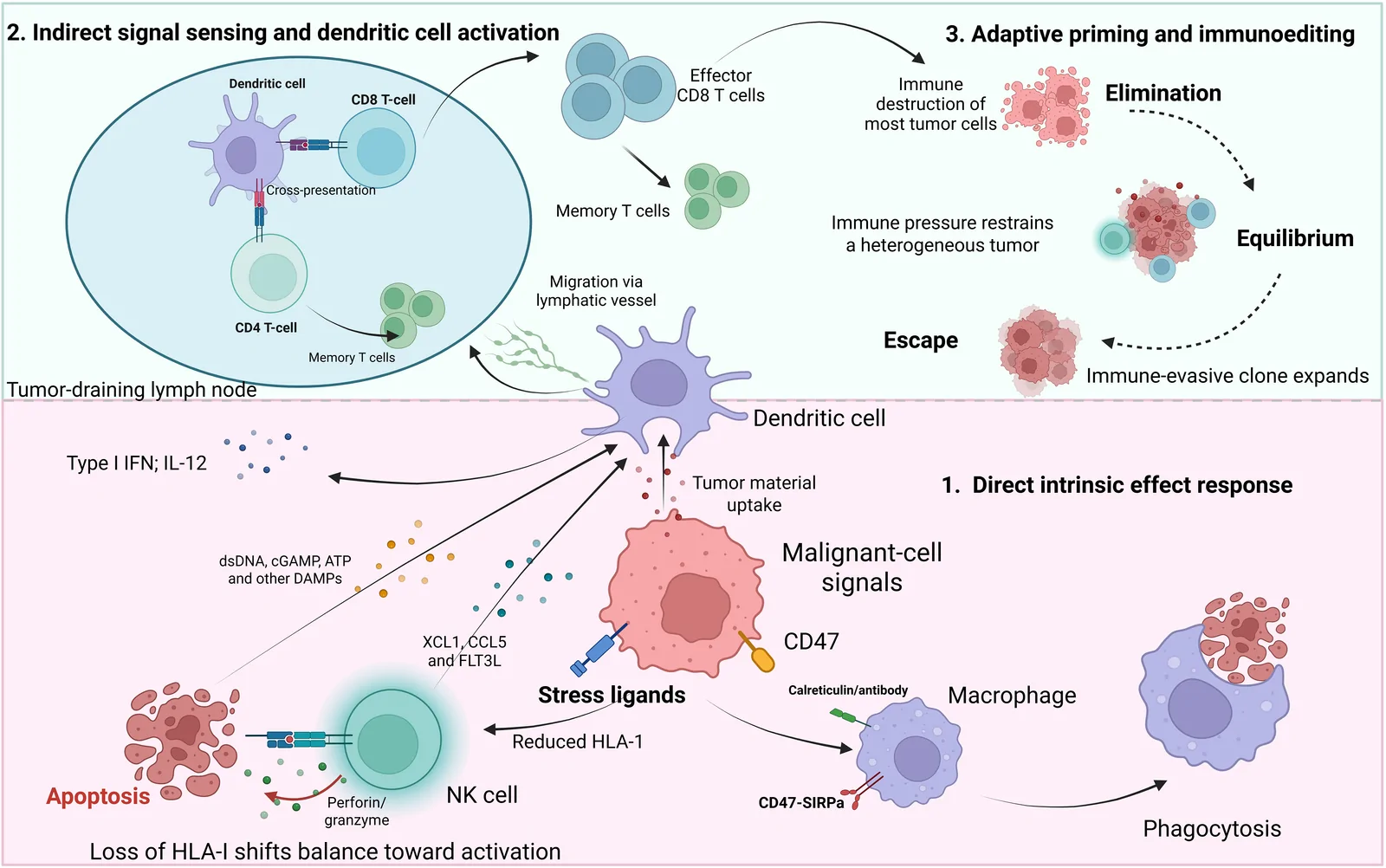
Integrated innate recognition, DC priming and cancer immunoediting. Innate tumor surveillance proceeds through complementary direct-recognition and indirect-sensing mechanisms. Reduced HLA-I expression and transformation-associated stress ligands shift the balance of NK-cell signaling toward activation, resulting in perforin–granzyme-mediated tumor-cell apoptosis. Macrophages integrate pro-phagocytic signals, including calreticulin exposure and antibody opsonization, with inhibitory CD47–SIRPα signaling to determine tumor-cell engulfment. In parallel, dsDNA, cGAMP, ATP and other DAMPs released by stressed or dying malignant cells activate endosomal TLRs and cytosolic cGAS–STING signaling in cDC1s, inducing type I IFN and IL-12 production. NK-cell-derived XCL1, CCL5 and FLT3L recruit and support cDC1s, whereas cDC1-derived CXCL9 promotes the positioning and recruitment of CD8^+^ T cells. Following antigen uptake, CCR7^+^ cDC1s migrate through lymphatic vessels to tumor-draining lymph nodes, where they cross-present tumor antigens to CD8^+^ T cells and present associated antigens through MHC-II to CD4^+^ T cells. CD40–CD40L-dependent licensing reinforces effector expansion and memory formation. The resulting immune pressure eliminates susceptible malignant cells but simultaneously selects resistant variants, driving progression from elimination through equilibrium to immune escape. ATP, adenosine triphosphate; CCL5, C-C motif chemokine ligand 5; cDC1, conventional type 1 dendritic cell; cGAMP, cyclic GMP–AMP; cGAS, cyclic GMP–AMP synthase; CXCL9, C-X-C motif chemokine ligand 9; FLT3L, FMS-like tyrosine kinase 3 ligand; HLA-I, human leukocyte antigen class I; IFN, interferon; IL-12, interleukin-12; MHC-II, major histocompatibility complex class II; NK, natural killer; SIRPα, signal-regulatory protein α; STING, stimulator of interferon genes; TLR, Toll-like receptor; XCL1, X-C motif chemokine ligand 1. Created in BioRender. Liu, D. (2026) https://BioRender.com/wi8pnuh.

## 3 Tumor-microenvironmental reprogramming of innate immunity and treatment response

Although frequently described as “tumor-derived immunosuppression,” this circuitry comprises both factors released directly by malignant cells and secondary suppressive programs induced in stromal and innate immune cells. TGF-β and VEGF exemplify this distributed organization: in metastatic urothelial cancer, a fibroblast TGF-β-response signature marked T-cell-excluded tumors and resistance to atezolizumab, whereas combined TGF-β and PD-L1 blockade promoted lymphocyte penetration and tumor regression in mouse models; independently, VEGF–VEGFR2 signaling induced PD-L1 and arginase-1 in tumor-associated myeloid cells, and its inhibition restored DC maturation, T-cell activation and checkpoint-blockade efficacy in breast and colorectal tumor models (22, 23). These soluble circuits intersect with metabolic competition: rapid nutrient consumption and defective perfusion restrict oxygen and nutrient availability while promoting acidosis and lactate accumulation; mechanistically, glioblastoma-derived factors induced GLUT1 + glycolytic monocyte-derived macrophages in which lactate-dependent histone lactylation drove IL-10 expression and T-cell suppression, whereas spatial profiling of human tumors localized adverse SPP1 + macrophages to hypoxic and necrotic regions (24, 25). Tumors also communicate through extracellular vesicles and endogenous nucleic-acid sensing: paclitaxel or doxorubicin elicited annexin-A6-enriched tumor vesicles that activated endothelial NF-κB–CCL2 signaling and expanded Ly6C +CCR2 + monocytes in the pulmonary premetastatic niche, whereas chromosomal instability generated chronic tumor-cell cGAS–STING signaling, type-I-interferon tachyphylaxis and ER-stress rewiring that established an immune-suppressive, prometastatic microenvironment (26, 27). Dysregulated chemokines then convert molecular suppression into selective cellular trafficking: elevated baseline serum IL-8 was associated with increased tumor neutrophils and poorer survival among 1,344 patients receiving nivolumab-based therapy, although its association with poor outcomes in non-immunotherapy arms indicates that IL-8 is presently better regarded as a marker of unfavorable neutrophilic tumor biology than as a treatment-specific predictive biomarker (28). Thus, immune exclusion is an actively constructed ecological state in which vascular dysfunction, stromal barriers, suppressive metabolites and chemokine gradients jointly favor regulatory myeloid populations while limiting access and function of NK cells and tumor-reactive lymphocytes.

The resulting myeloid compartment cannot be represented adequately by binary M1/M2 or N1/N2 classifications; instead, macrophages, monocytes, DCs and neutrophils occupy context-defined states whose function depends on ontogeny, metabolic substrate, treatment history and spatial neighborhood. Spatially resolved analysis of human breast and colon tissues identified five recurrent macrophage niches with divergent activities—IL4I1 + macrophages phagocytosed dying cells and predicted favorable outcome, SPP1 + macrophages occupied hypoxic–necrotic regions and predicted poorer outcome, and NLRP3 + macrophages colocalized with neutrophils and displayed inflammasome activity—illustrating why marker abundance without positional information can be misleading (25). Treatment-induced inflammation is correspondingly bifunctional. Longitudinal biopsies from triple-negative breast cancer showed that some initially immune-poor tumors developed productive antigen-presenting-myeloid-cell–cytotoxic-T-cell interactions only after pembrolizumab plus radiotherapy, whereas, in patients with glioblastoma, chemoradiotherapy-associated lymphopenia correlated with expanded circulating MDSCs, with causal and pharmacologically reversible effects demonstrated in mice (29, 30). Cytotoxic therapy can likewise initiate relapse-promoting repair: chemotherapy-associated release of nuclear IL-1α recruited and reprogrammed suppressive myeloid cells, and IL-1α neutralization improved paclitaxel and PD-1-blockade efficacy in preclinical models, while chemotherapy-elicited extracellular vesicles promoted monocyte-dependent metastatic colonization (26, 31). Finally, the biological outcome of innate sensing depends on signal duration and cellular state. Chronic tumor-cell STING activation can support immune suppression and dissemination, yet restoration of STING in reawakening lung-adenocarcinoma cells enabled their T-cell- and NK-cell-dependent elimination, and systemic STING agonism prevented metastatic outbreaks in mice (27, 32). Innate immunity therefore functions neither as a uniformly antitumor barrier nor as a fixed suppressive compartment, but as a plastic, treatment-responsive ecosystem whose spatial and temporal configuration determines therapeutic response, metastatic competence and the persistence of dormant residual disease.

In summary, tumor-derived suppressive signals remodel spatially and functionally heterogeneous innate immune populations, creating distinct opportunities for cell-selective therapeutic intervention (**Figure 2**).

**Figure 2:**
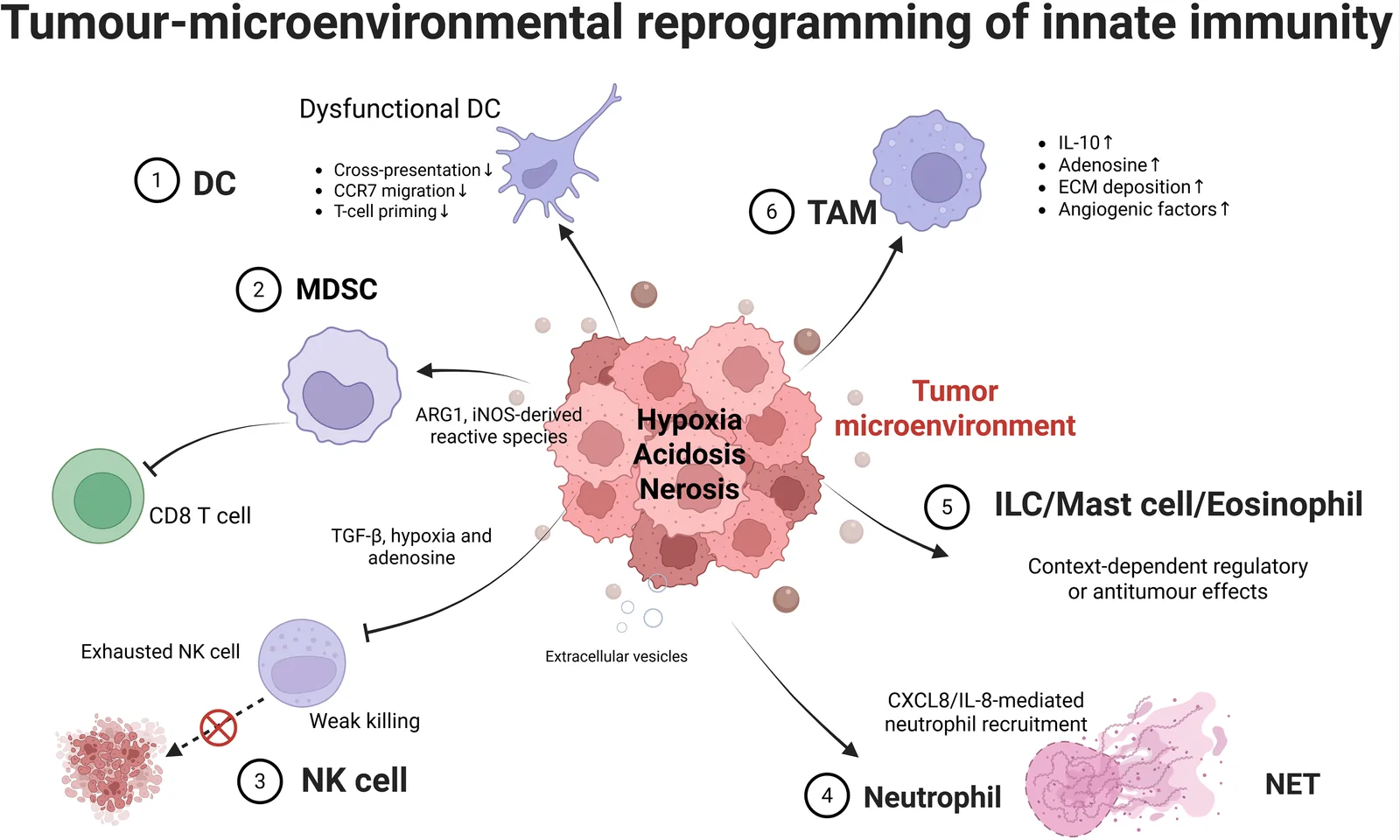
Tumor-microenvironmental reprogramming of innate immunity. Hypoxia, acidosis and aberrant vascularization establish a metabolically and spatially heterogeneous tumor microenvironment in which malignant and stromal cells release TGF-β, IL-10, VEGF, lactate, adenosine, extracellular vesicles, tumor-derived DNA and dysregulated chemokines. These signals impair DC maturation and antigen presentation, promote SPP1^+^ immunosuppressive tumor-associated macrophages, expand monocytic and polymorphonuclear MDSCs, suppress NK-cell cytotoxicity and recruit neutrophils capable of forming Neutrophil extracellular traps (NETs). MDSCs restrict CD8^+^ T-cell activity through arginase 1 (ARG1), inducible nitric-oxide synthase (iNOS)-derived reactive species and adenosine-dependent metabolic suppression, whereas CCL2 and CXCL8/IL-8 reinforce monocyte and neutrophil recruitment. ILCs, mast cells and eosinophils exert context-dependent regulatory or antitumor effects according to tumor type, tissue compartment and inflammatory state. Collectively, these programs promote T-cell exclusion, angiogenesis, extracellular-matrix remodelling, metastatic dissemination, tumor-cell dormancy or reawakening and resistance to systemic therapy. ARG1, arginase 1; CXCL8, C-X-C motif chemokine ligand 8; DC, dendritic cell; ECM, extracellular matrix; IL, interleukin; iNOS, inducible nitric oxide synthase; MDSC, myeloid-derived suppressor cell; NET, neutrophil extracellular trap; NK, natural killer; ROS, reactive oxygen species; TAM, tumor-associated macrophage; VEGF, vascular endothelial growth factor. Created in BioRender. Liu, D. (2026) https://BioRender.com/ksdj6d8.

## 4 DCs: antigen presentation and therapeutic exploitation

DCs link tumor sensing to adaptive immunity through three core functions: antigen uptake, processing and MHC presentation, and costimulatory licensing of T cells.

### 4.1 DC lineages and functional states in cancer

DC function in cancer is determined jointly by ontogeny and activation state. BATF3–IRF8-dependent XCR1^+^CLEC9A^+^ conventional type 1 DCs (cDC1s) are specialized for acquiring cell-associated antigens, cross-presentation and CD8^+^ T-cell priming, whereas CD1c^+^SIRPα^+^ cDC2s preferentially coordinate MHC-II-restricted CD4^+^ T-cell immunity; notably, experimental and human melanoma analyses showed that cDC2-mediated protection becomes most apparent when regulatory-T-cell pressure is limited, linking a high cDC2:Treg ratio to conventional CD4^+^ T-cell activation, survival and responsiveness to PD-1 blockade (33, 34). These lineage-associated specializations are not immutable. After tumor-antigen uptake, both cDC1s and cDC2s can converge on a CCR7^+^LAMP3^+^ mature regulatory dendritic-cell (mregDC) state that simultaneously expresses CD40 and IL-12 alongside PD-L1, PD-L2 and CD200. In mouse and human lung cancers, AXL promoted PD-L1 expression, whereas IL-4 constrained IFNγ-dependent IL-12 production. These findings demonstrate that phenotypic maturation does not necessarily confer productive immunogenicity (33).

Plasmacytoid DCs (pDCs) contribute principally through nucleic-acid sensing and type-I-interferon production, a pathway explored clinically with the TLR9 agonist vidutolimod: combined intratumoral vidutolimod and pembrolizumab produced durable responses in 25% of 44 patients with anti-PD-1-refractory metastatic melanoma, while neoadjuvant vidutolimod–nivolumab achieved a 55% major pathological response rate in 31 patients with high-risk resectable melanoma, with response associated—but not proven to be caused—by intratumoral pDC, myeloid-cell and CD8^+^-T-cell enrichment (35, 36). The human CD1c^+^ compartment additionally contains CD163^+^ DC3s, which arise through a GM-CSF-responsive pathway distinguishable from conventional-DC- and monocyte-restricted progenitors and can prime naïve CD8^+^ T cells toward a CD103^+^ tissue-resident phenotype, underscoring the inadequacy of assigning all CD1c^+^ APCs to cDC2 (37). Likewise, inflammatory monocyte-derived DCs and ex-vivo-generated moDC vaccines are ontogenetically and functionally distinct from cDCs and often display limited persistence, migration and antigen-presentation capacity after transfer; other myeloid cells can also present tumor antigens, but this is not invariably beneficial, as antigen presentation by IRF8^+^ tumor-associated macrophages(TAMs) promoted intratumoral cytotoxic-T-cell exhaustion rather than lymph-node priming in breast-cancer models (38, 39).

### 4.2 Antigen acquisition, cross-presentation and T-cell priming

Antitumor presentation begins with receptor-mediated endocytosis or phagocytosis of soluble proteins, extracellular vesicles and dying-cell material, followed by endolysosomal processing for MHC-II presentation or diversion of selected exogenous antigens into MHC-I cross-presentation pathways. The biochemical quality of this processing is decisive: lysosomal prosaposin and its saposin products facilitate disintegration and cross-presentation of membrane-associated apoptotic-body antigens, whereas tumor-derived TGFβ induced prosaposin hyperglycosylation and secretion, depleted lysosomal saposins and impaired CD8^+^ T-cell activation in mouse tumors and melanoma-associated human DCs (40).

Antigen trafficking is also a multicellular process rather than a property of a single DC: intravital imaging showed that migratory DCs transport tumor-antigen-containing vesicles to draining lymph nodes and transfer them through contact-dependent synapses to resident DC subsets, with vesicle-bearing—but not vesicle-negative—DCs acquiring T-cell-stimulatory capacity (41). Following antigen acquisition, CCR7 directs activated DCs toward CCL19/CCL21-rich tumor-draining lymph nodes; photoconversion-based tracking nevertheless revealed divergent fates, with CCR7^+^ DCs constituting the predominant tumor-derived myeloid emigrants while a tumor-retained CCR7^+^ population progressively lost antigen-presentation and pro-inflammatory transcriptional programs as its intratumoral dwell time increased (42). Productive priming therefore requires the coordinated delivery of peptide–MHC, CD80/CD86–CD28 costimulation, CD40-mediated licensing and polarizing cytokines such as type-I interferons and IL-12; proximity-based single-cell labeling demonstrated that only a small, hyperactivated fraction of DCs accounted for most cognate tumor-antigen presentation to CD4^+^ and CD8^+^ T cells in lymph nodes and tumors, and that checkpoint blockade intensified these CD40-associated DC–T-cell interactions (33, 43). These principles motivate strategies that expand, load and activate DCs *in situ*. A clinical Flt3L–radiotherapy–poly-ICLC regimen recruited cross-presenting intratumoral DCs and generated systemic CD8^+^ T-cell responses and regressions in advanced indolent lymphoma, whereas IL-12/FLT3L-engineered DC progenitors generated cDC1s, expanded endogenous DCs and controlled several murine tumors without predefined antigen loading, although the latter approach remains preclinical (38, 44).

### 4.3 Tumor-driven DC dysfunction

Tumor-induced DC dysfunction comprises coordinated defects in lineage maintenance, maturation, antigen processing and tissue trafficking, such that DCs may capture malignant material yet fail to generate stable peptide–MHC complexes, upregulate costimulatory molecules or productively activate tumor-specific lymphocytes; this state can be imposed locally by tumor-conditioned factors and systemically through depletion or tolerogenic reprogramming of cDC1s (45, 46). The shared metabolic and cytokine barriers that impair DC function—including TGF-β, VEGF, lactate and hypoxia—are discussed in the broader context of tumor-microenvironmental suppression in Section 3; here, we focus on DC-specific mechanisms. Soluble suppression extends beyond canonical cytokines: melanoma-secreted midkine acts through STAT3 across tumors, lymph nodes and bone marrow to impair DC differentiation, activation and antigen-presenting function, and an associated DC signature identified patients with poor prognosis and checkpoint-blockade resistance (46). Regulatory cells additionally impose a spatial barrier to DC migration; in colorectal tumors, CCR4-dependent recruitment of regulatory T cells to perilymphatic mregDCs generated a reciprocal niche that activated Tregs while restraining mregDC migration and antigen trafficking to draining lymph nodes, and disruption of this interaction inhibited tumor progression (47).

### 4.4 Therapeutic activation and engineering of DCs

Therapeutically, DC-directed interventions can be organized around four complementary objectives—supplying functional DCs ex vivo, expanding them *in vivo*, delivering antigen to lineage-restricted receptors and providing maturation signals while simultaneously removing downstream suppressive circuits. A personalized, neoantigen-loaded autologous DC vaccine tested in resected non-small-cell lung cancer (NSCLC) was manufacturable for six of ten enrolled patients and induced systemic responses in five of six recipients that remained detectable for up to 19 months; nevertheless, the randomized phase III MIND-DC study in 148 patients with resected stage IIIB/C melanoma showed that tumor-antigen-loaded CD1c^+^ conventional DCs plus plasmacytoid DCs increased functional antigen-specific T-cell responses from 3.8% to 67.1% without improving two-year recurrence-free survival, illustrating that vaccine immunogenicity is not equivalent to clinical efficacy (48, 49). An alternative is to manipulate endogenous DCs: in a randomized phase II melanoma study, FLT3 ligand expanded circulating cDC1s, cDC2s and plasmacytoid DCs, while an anti-DEC-205–NY-ESO-1 fusion vaccine targeted antigen to CD205 and poly-ICLC supplied TLR3-dependent activation, together increasing cellular and humoral antigen-specific immunity (50). CD40 agonism can license APCs and cooperate with antigen release, but clinical activity remains context dependent—the randomized PRINCE trial in metastatic pancreatic cancer did not meet its one-year overall-survival endpoint for either sotigalimab plus chemotherapy or the sotigalimab–nivolumab–chemotherapy triplet, although survival with sotigalimab correlated with intratumoral CD4^+^ T cells and circulating APCs, supporting biomarker-guided development (51). Checkpoint combinations should likewise be selected mechanistically: immunogenic DC vaccines induced suppressive PD-L1^+^ macrophage niches in lymph nodes and tumors, and PD-L1—but not PD-1—blockade restored vaccine-mediated tumor regression in mouse models; analogous expansion of PD-L1^+^ tumor-associated macrophages was detected after DC vaccination in patients with glioblastoma (52). Finally, fractionated radiotherapy was used preclinically to uncage a TLR7/8-agonist prodrug selectively within tumors, activating DC–T-cell circuits and abscopal immunity through convergent STING–MyD88–type-I-interferon signaling; however, early ferroptotic tumor cells instead reduced DC maturation, engulfment and cross-presentation and even compromised the vaccination activity of immunogenic apoptotic cells, emphasizing that the modality and timing of therapy-induced cell death determine whether antigen release becomes an *in situ* vaccine or an additional tolerogenic signal (53, 54).

## 5 TAMs: ontogeny, function and therapeutic reprogramming

TAMs illustrate how a single lineage can perform opposing functions—phagocytic tumor clearance in some niches and profibrotic, T-cell-excluding suppression in others—depending on ontogeny, spatial localization and metabolic conditioning.

### 5.1 Ontogeny and functional heterogeneity of TAMs

Tumor-associated macrophages (TAMs) do not constitute a single terminally polarized population, but arise from developmentally distinct tissue-resident macrophages and continuously recruited bone-marrow-derived monocytes whose relative abundance, localization and phenotype change during tumor evolution (55, 56). In autochthonous NSCLC, lineage tracing showed that self-maintaining resident macrophages initially accumulated beside transformed epithelial cells, induced epithelial–mesenchymal transition and invasiveness, and supported regulatory T-cell responses; as lesions progressed, these cells relocated to the tumor margin while monocyte-derived macrophages became numerically dominant, a temporal organization also discernible in human tumors (55). Complementary fate mapping in pancreatic ductal adenocarcinoma demonstrated that recruited monocytes first passed through a transient CCR2^+^CD64^+^ state before generating spatially segregated, longer-lived populations, including Maf-regulated CD73^+^ macrophages in the hypoxic core and lipid-handling TREM2^+^ macrophages outside it, with analogous populations detected in human disease (56). At broader resolution, single-cell profiling of myeloid cells from 210 patients across 15 cancer types uncovered marked tumor-specific variation in myeloid composition and in the markers used by pro-angiogenic TAMs, whereas analysis of 52 head-and-neck cancers identified a spatially organized CXCL9–SPP1 macrophage-polarity axis that coordinated wider pro- or antitumor cellular programs and was more prognostically informative than conventional M1/M2 signatures (57, 58). These findings expose the central limitation of binary M1/M2 nomenclature: individual TAMs can simultaneously express inflammatory, reparative, metabolic and suppressive modules, and canonical markers need not predict function—for example, conditional depletion of CD206^+^ macrophages removed CXCL9-producing cells, disrupted a cDC1–NK–CD8^+^ T-cell axis and impaired antigen-specific tumor control in mice, while a corresponding CD206-replete human signature was associated with improved survival (58, 59). TAM states should therefore be defined through the integration of ontogeny, anatomical niche, transcriptional and metabolic programs, and experimentally demonstrated function—not through isolated “M1” or “M2” markers.

### 5.2 Immunosuppressive, stromal and metastatic functions of macrophages

Once incorporated into tumor-supportive niches, selected macrophage states suppress immunity both biochemically and spatially: TAMs form unusually persistent, antigen-dependent but poorly activating contacts with CD8^+^ T cells, and reciprocal macrophage–T-cell signaling establishes an intratumoral positive-feedback circuit that promotes T-cell exhaustion; accordingly, TAM depletion reduced exhaustion programs and restored effector potential in mouse tumors (60). Macrophages also cooperate with stromal cells to construct immune-excluded tissue architecture: in colorectal cancer, spatially colocalized SPP1^+^ macrophages and FAP^+^ fibroblasts formed a TGF-β/IL-1-associated desmoplastic network enriched for collagen- and matrix-remodelling programs, surrounding malignant regions from which lymphocytes were excluded (61). Mechanistically, the stiffened breast-tumor matrix can induce a TGF-β-dependent collagen-biosynthesis program in TAMs that consumes extracellular arginine, generates proline and releases ornithine, thereby coupling fibrosis to metabolic restriction of CD8^+^ T-cell function (62). Pro-angiogenic activity is likewise state- and tumor-dependent; Notably, lipid-droplet-loaded macrophages in human glioblastoma acquired hypoxia-, mesenchymal-transition- and angiogenesis-associated features after scavenging lipids from tumor extracellular vesicles; their abundance correlated with adverse outcome, and inhibition of lipid-droplet-forming enzymes disrupted this state (57, 63).

Macrophage-derived signals additionally act directly on malignant cells: lung-resident macrophages induce invasiveness during early tumorigenesis, whereas intravital imaging of breast cancer revealed that macrophages surrounding intravasation sites prime disseminating cells for invasion, rapid extravasation, survival and dormancy, potentially linking primary-tumor conditioning to delayed recurrence (55, 64). Direct macrophage–tumor-cell contact can also activate Notch–Jagged signaling and induce a stem-like phenotype near tumor microenvironment of metastasis doorways; these cells became strongly enriched among circulating tumor cells, and Notch inhibition reduced pulmonary metastatic burden, providing a mechanistic route to stemness-associated dissemination and chemoresistance (65). Clinically, high SPP1/FAP programs were associated with inferior anti-PD-L1 outcomes, whereas macrophage-generated fibrotic and metabolic barriers provide causal preclinical mechanisms through which such niches may limit checkpoint therapy (61, 62). Proof-of-concept translation has come from the phase I/II MATINS study: CLEVER-1 blockade with bexmarilimab produced no dose-limiting toxicity in the 30-patient escalation phase, showed 25–40% disease-control rates in selected expansion cohorts, and induced macrophage, IFN-γ and T-cell-receptor programs in patients attaining disease control, although these uncontrolled signals require confirmation in randomized studies (66).

### 5.3 Therapeutic depletion, reprogramming and engineering of macrophages

Macrophage-directed interventions encompass three conceptually distinct strategies—reducing macrophage abundance, preventing monocyte recruitment and reprogramming TAMs—whose therapeutic indices depend on preserving antitumor and tissue-resident subsets while eliminating tumor-supportive states. The clearest clinical validation of CSF1-CSF1R dependence comes from the phase III ENLIVEN trial in CSF1-driven tenosynovial giant-cell tumors. Pexidartinib produced a 39% RECIST response rate at week 25 versus 0% with placebo. However, potentially severe mixed or cholestatic hepatotoxicity illustrates the liabilities of systemic CSF1R inhibition and the limited generalizability of this unusually macrophage-dependent malignancy (67). Moreover, murine gliomas recurring during CSF1R inhibition acquired a microenvironmentally mediated, reversible resistance state sustained by macrophage-derived IGF1 and tumor-cell IGF1R–PI3K signaling, indicating that macrophage depletion or suppression can select compensatory trophic circuits rather than produce durable eradication (68). Recruitment blockade offers an alternative: in a phase Ib study of borderline-resectable or locally advanced pancreatic cancer, the CCR2 inhibitor PF-04136309 combined with FOLFIRINOX reduced CCR2-positive inflammatory monocytes and intratumoral macrophage accumulation and yielded an objective response in 16 of 33 evaluable patients, although the small, non-randomized design precluded attribution of efficacy to CCR2 inhibition (69). Because CSF1R and CCL2–CCR2 interventions may incompletely distinguish deleterious from protective macrophages, state-selective reprogramming is increasingly favored: genetic deletion or antibody blockade of TREM2 in mouse tumors depleted MRC1-positive suppressive macrophage states, expanded inflammatory myeloid populations and potentiated PD-1 blockade without requiring wholesale macrophage ablation (70).

A complementary strategy releases or actively redirects macrophage effector functions. Blockade of the CD47–SIRPα “don’t-eat-me” checkpoint lowers the phagocytic threshold, and the inactive-Fc CD47 decoy evorpacept demonstrated manageable hematological toxicity in the first-in-human ASPEN-01 trial, with preliminary response rates of 20% with pembrolizumab in head-and-neck cancer and 21.1% with trastuzumab in gastric or gastro-oesophageal-junction cancer; these uncontrolled signals nevertheless require randomized confirmation (71). Agonistic CD40 signaling can simultaneously license APCs and remodel macrophages: neoadjuvant selicrelumab in 16 patients with resectable pancreatic cancer was associated with increased mature DCs and activated T cells, reduced fibrosis and fewer M2-like macrophages relative to untreated or chemotherapy-treated specimens, although the study was designed for biological rather than efficacy assessment (72). Intratumoral innate agonists have likewise established biological activity but uneven clinical efficacy. The TLR9 agonist vidutolimod plus pembrolizumab produced objective responses in 11 of 44 patients with PD-1-resistant melanoma, whereas the STING agonist MIW815 plus spartalizumab generated on-target immune activation but only a 10.4% response rate across 106 patients with advanced cancers (35, 73). Spatially restricted delivery may improve this therapeutic window: cyclodextrin nanoparticles carrying the TLR7/8 agonist R848 preferentially accumulated in TAMs, induced inflammatory repolarization and enhanced PD-1 blockade in mouse tumor models while limiting systemic exposure (74). Extending this principle to durable cellular programming, macrophage-tropic lentiviral delivery of interferon-α reshaped antigen presentation and T-cell recruitment and eradicated experimental liver metastases, although emergent IL-10- and CTLA-4-associated regulatory circuits underscored the plasticity of the engineered microenvironment (75). Finally, induced-pluripotent-stem-cell-derived PSCA-directed chimeric antigen receptor macrophages incorporating membrane-bound IL-15 and an EGFR safety switch showed potent activity in pancreatic-cancer models. The first-in-human HER2 CAR-macrophage trial established trafficking, microenvironmental remodeling and an encouraging safety profile but no objective responses among 14 patients. These findings position engineered macrophages as a feasible yet experimentally immature platform for solid-tumor immunotherapy (76, 77).

## 6 NK cells: tumor recognition, dysfunction and therapeutic engineering

NK cells are the principal innate effector lymphocytes that integrate direct activating and inhibitory receptor signals to execute immediate cytotoxicity, while simultaneously shaping adaptive responses through chemokine secretion and DC crosstalk.

### 6.1 Receptor integration and effector mechanisms of NK-cell tumor recognition

NK-cell activation is determined by the integrated strength, duration and spatial organization of signals from inhibitory receptors—principally killer-cell immunoglobulin-like receptors recognizing classical HLA-I and CD94–NKG2A recognizing HLA-E—and activating receptors including NKG2D, DNAM-1 and the natural-cytotoxicity receptors NKp30, NKp44 and NKp46. Accordingly, “missing-self” recognition occurs when malignant cells lose HLA-I-mediated inhibitory input, whereas “induced-self” recognition reflects the acquisition of stress-associated ligands such as MICA, MICB and ULBPs; antibody blockade of NKG2A established the HLA-E–NKG2A axis as a druggable NK- and T-cell checkpoint, while antibodies stabilizing surface MICA/B demonstrated that preserving induced-self signals restores NK-dependent tumor control in mice (78, 79).

Once the activation threshold is exceeded and an immune synapse forms, perforin enables granzyme delivery into the target-cell cytosol, whereas Fas ligand–Fas and TRAIL–TRAIL-receptor interactions provide granule-independent apoptosis; live-cell imaging showed that serially killing NK cells preferentially use rapid granzyme-B-mediated death during early encounters but switch toward slower death-receptor killing as perforin and granzyme stores decline and surface Fas ligand increases (80). NK cells additionally secrete IFN-γ and TNF and produce chemokines such as XCL1, CCL3 and CCL5, thereby suppressing tumor proliferation, activating myeloid cells and coordinating subsequent adaptive immunity rather than functioning solely as autonomous killers. CD16A-mediated recognition of antibody-opsonized targets provides a distinct antigen-specific entry point. In the randomized phase III SOPHIA trial, the Fc-engineered anti-HER2 antibody margetuximab binds activating CD16A more strongly and inhibitory CD32B less strongly than trastuzumab. It produced a modest improvement in median progression-free survival over trastuzumab plus matched chemotherapy (5.8 versus 4.9 months). The trial could not isolate NK-cell antibody-dependent cellular cytotoxicity from other antibody and chemotherapy effects (81). More direct CD16A redirection was achieved by precomplexing cord-blood NK cells with the CD30×CD16A engager AFM13: a phase I study in 42 heavily pretreated CD30-positive lymphomas reported overall and complete response rates of 92.9% and 66.7%, respectively, without cytokine-release syndrome, neurotoxicity or graft-versus-host disease, although lymphodepletion, repeated AFM13 dosing and the single-arm design limit causal and comparative interpretation (82).

### 6.2 Tumor-driven NK-cell dysfunction and immune escape

In established tumors, NK-cell failure is better viewed as a continuum of rapidly acquired, context-dependent dysfunction than as a unitary, terminally exhausted state: in mouse tumors, NK cells quickly lost degranulation and cytokine competence after entry and adopted tissue-residency-associated features, whereas IL-15 preserved effector function and restored tumor control, demonstrating that at least part of this phenotype remains reversible (15). Human single-cell atlases reinforce this heterogeneity: analysis of 716 patients across 24 cancers identified tumor-enriched NK states with reduced cytotoxic programs and associations with adverse outcome and immunotherapy resistance, while reference mapping of 427 patients with seven solid-tumor types distinguished a stressed, TME-sensitive CD56 bright state from a relatively resistant cytotoxic CD56 dim state whose ratio predicted outcome in melanoma and osteosarcoma (83, 84). These datasets also suggest that chemokine-receptor mismatches preferentially recruit or retain CXCR3-positive CD56 bright cells while limiting access of mature CD56 dim effectors, although transcriptomic and spatial associations alone do not establish defective trafficking as the causal determinant of NK-cell exclusion (84). Metabolic suppression converges with altered localization and compounds the rapid post-entry loss of effector competence and chemokine-receptor mismatches that restrict the recruitment and function of mature CD56dim NK cells (15, 84). Finally, tumors can remove the very signals required for recognition: proteolytic MICA/B shedding reduces membrane NKG2D engagement and generates soluble ligands capable of perturbing NK function, whereas acute-myeloid-leukemia stem cells selectively lacking NKG2D ligands resist NK-cell clearance and can be sensitized by pharmacological induction of ligand expression (79, 85). Thus, effective restoration of NK surveillance will probably require simultaneous correction of trafficking, metabolic fitness and receptor–ligand availability rather than amplification of cytotoxicity alone.

### 6.3 Cytokine, engager and cellular NK-cell therapies

NK-cell therapeutics are converging on modular approaches that increase activating signals, release inhibitory checkpoints or physically couple NK cells to malignant targets. Although high-dose IL-2 can expand and activate NK cells, concomitant stimulation of CD25 high regulatory T cells and substantial systemic toxicity restrict its therapeutic window; the *de novo*-designed Neo-2/15 agonist circumvents IL-2Rα and IL-15Rα while engaging IL-2Rβγc, achieving greater tumor control and lower toxicity than IL-2 in murine melanoma and colon-cancer models (86). IL-15 more selectively supports NK-cell and memory-CD8 + T-cell proliferation, and trans-presentation-inspired agonists such as the IL-15N72D–IL-15Rα/Fc complex N-803 (formerly ALT-803) offer prolonged pharmacological activity; in a 21-patient phase Ib NSCLC study, N-803 plus nivolumab caused no dose-limiting toxicities and produced six partial responses, although the single-arm design precluded attribution of efficacy specifically to IL-15 stimulation (87). Checkpoint-directed strategies seek to interrupt inhibitory KIR, NKG2A, TIGIT or related receptor pathways without inducing chronic, non-selective activation: notably, TIGIT expression marked dysfunctional NK cells in tumor-bearing mice and human colorectal-cancer specimens, whereas genetic deletion or antibody blockade restored NK-cell-dependent tumor control and promoted protective antitumor memory in preclinical models (88). Tumor-targeting monoclonal antibodies meanwhile exploit CD16-mediated antibody-dependent cellular cytotoxicity, but Fc competition, antigen density and CD16 shedding can limit this mechanism; multispecific NK-cell engagers address these constraints by enforcing higher-avidity immune synapses, exemplified by a CD123×NKp46×CD16a engager that activated NK cells against primary acute myeloid leukemia (AML), outperformed an Fc-optimized anti-CD123 antibody *in vivo* and induced sustained CD123 + cell depletion in non-human primates without pronounced inflammatory cytokine release (89). These observations favor combination architectures in which cytokine agonism or checkpoint release increases NK-cell competence while antibodies and engagers confer tumor selectivity, ideally using tumor-conditional or locally retained molecules to prevent systemic activation and activation-induced dysfunction.

Adoptive NK-cell therapy further separates the effector-cell source from the immunosuppressive patient environment. Autologous products minimize donor incompatibility but may retain disease- and treatment-associated dysfunction, healthy-donor peripheral-blood or cord-blood NK cells enable allogeneic manufacture with little graft-versus-host potential, and clonally selected induced-pluripotent-stem-cell (iPSC) lines provide a renewable substrate for standardized, multiplex-engineered products. Brief IL-12/IL-15/IL-18 priming generates epigenetically imprinted memory-like NK cells with enhanced recall cytotoxicity; first-in-human AML testing demonstrated post-transfer expansion and antileukaemic function, and a subsequent study of donor-derived memory-like cells in post-transplant relapsed pediatric or young-adult AML achieved day-28 complete remission in four of eight evaluable patients, with functional persistence beyond three months and no significant treatment-related toxicity (90, 91). iPSC technology permits edits to be introduced once at the master-cell-clone stage: CRISPR-mediated CISH deletion augmented IL-15–JAK–STAT signaling, glycolytic and oxidative metabolism, *in-vivo* persistence and xenograft control by iPSC-derived NK cells, whereas the first-in-human FT596 study subsequently showed that an iPSC-derived product incorporating a CD19 CAR, non-cleavable high-affinity CD16 and an IL-15–IL-15R fusion could be repeatedly administered to 86 patients with B-cell lymphoma, with acceptable tolerability and deep, sometimes durable responses despite the absence of a randomized comparator (92, 93). Clinical CAR-NK evidence is strongest in B-cell malignancies: a 37-patient trial of cord-blood-derived CAR19/IL-15 NK cells reported identical day-30 and day-100 overall-response rates of 48.6%, without notable cytokine-release syndrome, neurotoxicity or graft-versus-host disease, while a separate eight-patient study of repeatedly dosed, IL-15-armoured 4-1BB–CD3ζ CAR-NK cells produced a 62.5% response rate and 50% complete-response rate, with persistence correlating with benefit (94, 95). Translation to solid tumors nevertheless remains predominantly preclinical because trafficking, extracellular-matrix exclusion, hypoxia, nutrient limitation and TGF-β-family signals jointly curtail persistence and serial killing; locoregionally delivered IL-21-engineered NK cells addressed part of this problem in glioblastoma models by activating a STAT1/STAT3–CEBPD program that preserved metabolic fitness and durable cytotoxicity with less toxicity than IL-15 armouring (96). Complementarily, SMAD4 knockout rendered peripheral-blood-, stem-cell-derived and CAR-NK platforms resistant to both TGF-β and activin A, preserving cytokine-driven proliferation, chemokine-receptor expression, tumor penetration and antibody-supported tumor control (97). Thus, next-generation solid-tumor products will probably require integrated chemokine-receptor matching or locoregional delivery, cytokine or metabolic armouring, resistance to suppressive ligands, enhanced persistence and multispecific recognition rather than escalation of any single activating module.

## 7 Neutrophil plasticity in cancer progression and therapy

Neutrophils in cancer are not fixed pro- or antitumor lineages but adopt context-dependent functional states, ranging from interferon-responsive populations that support T-cell activity to neutrophil extracellular trap (NET)-forming cells that promote immune exclusion, angiogenesis and metastatic awakening.

### 7.1 Heterogeneity and context-dependent states of tumor-associated neutrophils

Neutrophil heterogeneity in cancer is more accurately represented as a continuum shaped by maturation, anatomical compartment, tumor stage and therapy than by stable “N1” and “N2” lineages; accordingly, antitumor neutrophils, conventional tumor-associated neutrophils and polymorphonuclear MDSCs should be treated as context-dependent functional states rather than mutually exclusive ontogenies (98, 99). In orthotopic pancreatic tumors, integrated single-cell transcriptomic, epigenomic and proteomic analyses showed that immature CD101 – and mature CD101 + neutrophils entering from the circulation converged through tumor-local reprogramming into a terminal dcTRAIL-R1 + state concentrated in hypoxic, glycolytic tumor cores, where VEGFA production supported angiogenesis and tumor growth (98). Complementary profiling of peripheral-blood and tumor-infiltrating neutrophils from patients with pancreatic ductal adenocarcinoma identified inflammatory, transitional, interferon-responsive and terminally differentiated populations; the adverse-prognosis TAN-1 population was driven by hypoxia–endoplasmic-reticulum-stress-dependent BHLHE40 and hyperactivated glycolysis, which experimentally conferred immunosuppressive and protumor activity (99). Conversely, successful immunotherapy in murine lung- and colorectal-cancer models acutely expanded **Sell** hi, interferon-responsive neutrophils through a BATF3 + DC–IL-12–T-cell–IFNγ–IRF1 circuit, and a related systemic response correlated with clinical outcome in patients with lung cancer; independently, a baseline circulating interferon-stimulated neutrophil signature induced by tumor-intrinsic STING signaling predicted anti-PD-1 response in NSCLC and melanoma cohorts and functionally promoted IL-12-dependent cytotoxic-T-cell activity (100, 101). These observations also explain why circulating neutrophil abundance or the neutrophil-to-lymphocyte ratio cannot be assumed to report intratumoral function. For instance, recruitment through G-CSF/GM-CSF and CXCL1/2/5/8–CXCR2 pathways is followed by tissue-specific education by hypoxia, metabolites, TGF-β, interferons and stromal mediators; in the metastatic lung, for example, CD140a + mesenchymal-cell-derived PTGS2/PGE2 converted minimally suppressive circulating neutrophils into potent inhibitors of T and NK cells, whereas genetic or pharmacological interruption of PGE2 signaling reduced breast-cancer metastasis and improved adoptive T-cell therapy in mice (102). Importantly, this plasticity is therapeutically bidirectional. For instance, a preclinical combination of TNF, CD40 agonism and a tumor-binding antibody activated a complement C5a–leukotriene-B4–xanthine-oxidase/ROS cascade that enabled neutrophil-mediated clearance of several solid tumors, while T-cell immunotherapies recruited nitric-oxide-producing neutrophils to eliminate antigen-loss melanoma variants that had escaped direct T-cell recognition (20, 103).

### 7.2 Immunosuppression, NETosis and metastatic progression

Pathologically activated neutrophils promote cancer through overlapping programs of nutrient depletion, ARG1 and ROS production, inhibitory-ligand expression and suppression of cytotoxic lymphocytes, while their VEGFA, neutrophil elastase and MMP9 remodel vessels, basement membranes and ECM to facilitate invasion and angiogenesis (98, 102). NETs—decondensed chromatin decorated with histones, myeloperoxidase, elastase and matrix-degrading proteases—provide a particularly consequential extracellular effector mechanism. For instance, tumor-derived CXCR1/2 agonists induced NETosis in mouse and human neutrophils, and the resulting structures physically insulated cancer cells from CD8 + T-cell and NK-cell contact, whereas PAD4 or CXCR1/2 inhibition sensitized tumors to combined PD-1 and CTLA-4 blockade in preclinical models (104). In pancreatic cancer, IL-17 promoted neutrophil recruitment and NET formation through tumor-cell-derived factors. This established an immunosuppressive barrier that mediated resistance to checkpoint blockade. DNase I or pharmacological inhibition of NET formation restored T-cell infiltration and therapeutic responsiveness in mice (105). NETs also act as active metastatic guidance systems rather than passive meshes: NET-DNA accumulated in liver metastases from patients with breast and colorectal cancer and engaged the cancer-cell receptor CCDC25 to activate ILK–β-parvin signaling, enhance motility and attract disseminated cells into NET-rich liver and lung niches, while circulating NET markers predicted subsequent liver metastasis in an early-stage breast-cancer cohort (106). Systemic therapy can inadvertently amplify these programs. For instance, chemotherapy-treated breast-cancer cells released IL-1β and induced NETosis, after which NET-associated integrin αvβ1 captured latent TGF-β and MMP9 proteolytically activated it, driving epithelial-to-mesenchymal transition and resistance of lung metastases to chemotherapy (107). More recently, recombinase-based lineage tracing directly demonstrated that doxorubicin or cisplatin can reactivate dormant breast-cancer cells in the lung by inducing fibroblast senescence and secondary NET formation; NET-dependent extracellular-matrix remodelling promoted disseminated-cell proliferation, whereas combining chemotherapy with the senolytics dasatinib and quercetin suppressed reawakening and metastatic relapse in mice (108). Collectively, these findings argue against indiscriminate neutrophil depletion—which could remove antimicrobial and therapy-supporting populations—and instead favor state- and context-selective interventions targeting pathogenic recruitment, PGE2 or metabolic reprogramming, NET formation or individual NET effectors, ideally accompanied by paired blood–tumor profiling to preserve beneficial interferon-responsive and tumoricidal neutrophils.

### 7.3 Therapeutic targeting and exploitation of neutrophils

Therapeutic modulation of cancer-associated neutrophils should selectively intercept tumor-supporting recruitment and effector programs while preserving homeostatic granulopoiesis and antimicrobial competence. Along the trafficking axis, the dual CXCR1/2 inhibitor SX-682 reduced suppressive myeloid infiltration and potentiated docetaxel in HPV-negative head-and-neck cancer models, whereas simultaneous disruption of CCR1 and CXCR2 produced stronger suppression of colorectal tumor growth and liver metastasis than either intervention alone, indicating that compensatory chemokine circuits may limit single-receptor blockade (109, 110). Human evidence remains heterogeneous: in a 23-patient phase I study of androgen-receptor-signaling-inhibitor-resistant metastatic prostate cancer, AZD5069 plus enzalutamide reduced circulating neutrophils and intratumor CD11b^+^HLA-DR loCD15^+^CD14^-^ cells, with five of 21 evaluable patients meeting prespecified composite partial-response criteria, whereas randomized phase II testing of navarixin plus pembrolizumab across castration-resistant prostate cancer, microsatellite-stable colorectal cancer and NSCLC yielded an overall response rate of only 3% and was stopped for futility (111, 112). These findings argue that CXCR1/2 inhibition is not intrinsically tumoricidal and will probably require biomarker selection for chemokine-high, neutrophil-dependent disease, pharmacodynamic confirmation of intratumoral—not merely circulating—myeloid displacement, and combinations matched to the operative resistance mechanism. NET-directed therapy can instead prevent chromatin extrusion or dismantle established traps. For instance, the selective PAD4 inhibitor BMS-P5 blocked myeloma-induced NET formation and delayed disease in syngeneic models, while a near-infrared-II-responsive DNase-I nanoplatform enabled spatially gated NET degradation, improved checkpoint-blockade activity in colorectal tumors and reduced NET-mediated hepatic seeding (113, 114). A complementary CCDC25-decorated, DNase-I-loaded liposome exploited the affinity of CCDC25 for NET DNA to concentrate enzymatic activity in NET-rich niches and suppress colorectal-cancer liver metastasis in mice, illustrating how molecular targeting may reduce the exposure required for systemic DNase therapy (115); nevertheless, PAD4 inhibition, nuclease delivery and chemokine blockade remain investigational in oncology, with no established clinical efficacy against NET-dependent cancer.

Functional reprogramming offers a conceptually attractive alternative to indiscriminate depletion because it seeks to recover the cytotoxic potential of neutrophils already present in tumors. In breast-cancer models, TLR3 activation induced granule and NADPH-oxidase programs and, when combined with mitochondrial complex-I inhibition, amplified respiratory-burst-dependent tumor killing; independently, non-internalized adhesive polymer micropatches shifted transferred neutrophils toward an inflammatory antitumor state, activated systemic innate and adaptive immunity, and produced complete regression in one-third of B16F10-bearing mice when combined with CTLA-4 blockade (116, 117). Neutrophil chemotaxis can also be repurposed for drug transport: photoinduced local inflammation enabled neutrophils to carry high-payload paclitaxel nanocrystals across tumor endothelium and sustain intratumoral drug release, whereas a subsequent platform loaded neutrophils with doxorubicin nanoparticles and used localized inflammatory recruitment and NET-associated release to treat heterogeneous murine mammary tumors (118, 119). These carrier systems remain preclinical and present a mechanistic tension—efficient delivery requires intact neutrophil trafficking and activation, whereas CXCR1/2 inhibition or NET suppression may disable precisely those processes—making treatment sequencing, payload leakage, cell viability and off-target inflammatory deposition critical design variables. Safety signals from early CXCR2 trials are reassuring but not definitive: AZD5069 caused on-target neutropenia in 83% of patients, including grade ≥3 events in 48%, while neither that small study nor the navarixin trial identified a clear excess of serious infection or febrile neutropenia (111, 112). Importantly, NETs can enhance macrophage phagocytosis and bacterial killing by transferring neutrophil antimicrobial proteins, demonstrating that sustained systemic NET disruption could compromise cooperative host defense even when circulating neutrophil numbers are retained (120). Clinical translation should therefore favor tumor-restricted, reversible or intermittently dosed interventions, accompanied by neutrophil-count and infection surveillance and by assays that distinguish suppression of tumor-promoting states from loss of protective antimicrobial function.

## 8 MDSCs in tumor immune evasion

MDSCs are best understood not as a discrete lineage but as a convergence of suppressive effector programs—arginase, reactive oxygen/nitrogen species and adenosine—that are superimposed on polymorphonuclear and monocytic myeloid differentiation.

### 8.1 Definition, classification and phenotypic diversity of MDSCs

MDSCs are best conceptualized not as a discrete lineage, but as pathologically activated neutrophil- and monocyte-lineage states whose defining property is demonstrable immunosuppression; the principal classes are polymorphonuclear MDSCs (PMN-MDSCs) and monocytic MDSCs (M-MDSCs), with a phenotypically immature early-stage MDSC compartment additionally recognized in humans (121). Single-cell analysis of a murine breast-cancer model traced MDSCs through an aberrant splenic neutrophil-maturation trajectory and identified CD84 as an enrichment marker, whereas transcriptomic profiling of mature PMN-MDSCs from patients and granulocyte colony-stimulating factor-treated donors nominated CD52, CD84 and PTGER2 as a complementary human marker set (122, 123). Multimodal profiling subsequently resolved classical neutrophils, PMN-MDSCs and highly suppressive activated PMN-MDSCs in tumor-bearing mice, together with corresponding PMN states in patients whose tumor-associated signature correlated with adverse outcome, underscoring that suppressive activity represents a continuum superimposed on granulocytic differentiation (124). This context dependence is particularly evident in human glioma: single-cell and spatial profiling of 33 tumors identified metabolically adapted early-progenitor and monocytic MDSC populations in IDH-wild-type glioblastoma, with early-progenitor MDSCs concentrated in hypoxic pseudopalisading niches, illustrating why universal surface-marker definitions remain unlikely (125).

### 8.2 Molecular and metabolic mechanisms of MDSC-mediated suppression

MDSC-mediated suppression is mechanistically cooperative and spatially compartmentalized. In renal-cell carcinoma, activated granulocytic MDSCs released ARG1 and depleted extracellular l-arginine, thereby restricting T-cell responsiveness. MDSC-derived iNOS and nitric oxide inhibited DC antigen presentation to CD4^+^ T cells and suppressed T-cell proliferation in an iNOS-inhibitor-reversible manner (126, 127). NOX2-dependent ROS and iNOS-derived nitric oxide additionally feed a reactive-nitrogen-species network capable of generating peroxynitrite. In tumor models, nitration of CCL2 trapped tumor-specific T cells in the surrounding stroma while relatively preserving myeloid recruitment, whereas pancreatic-cancer surgery induced NOX2^+^ M-MDSC-like cells whose ROS suppressed natural-killer-cell function ex vivo and whose expansion predicted inferior postoperative survival (128, 129). Metabolic restriction extends beyond amino-acid starvation, as human MDSCs transferred methylglyoxal to CD8^+^ T cells and imposed a low-metabolic, functionally paralysed state, while tumor-educated M-MDSCs generated adenosine-dependent suppression that was relieved by PEGylated adenosine deaminase, sensitizing preclinical tumors to PD-1 blockade (130, 131). MDSCs also restructure the regulatory compartment. In tumor-bearing mice, CD40 expression on MDSCs was required for tumor-specific regulatory-T-cell generation and accumulation. It was also required for the establishment of T-cell tolerance (132). Thus, MDSCs suppress antitumor immunity simultaneously by limiting nutrients and metabolic fitness, chemically modifying receptors and chemokines, excluding effector T cells, expanding regulatory T cells, disabling DC priming and attenuating NK-cell cytotoxicity; the relative dominance of each pathway is dictated by MDSC subset, tissue niche, disease stage and therapeutic context.

### 8.3 Therapeutic targeting of MDSC development and function

Because MDSCs represent dynamically generated functional states rather than a fixed lineage, therapeutic strategies can intervene at four partially overlapping levels—pathological myelopoiesis, tumor recruitment, suppressive programming and survival—while requiring subset- and tissue-specific pharmacodynamic markers to distinguish genuine MDSC modulation from nonspecific myelotoxicity. At the production stage, genetic deletion or pharmacological inhibition of the NF-κB-family transcription factor c-Rel impaired murine and human MDSC development, extinguished suppressive gene programs and cooperated with PD-1-axis blockade in tumor models, whereas liver-X-receptor activation induced an ApoE–LRP8-dependent loss of MDSC survival, enhanced cytotoxic-lymphocyte activity in mice and reduced circulating MDSCs in an exploratory analysis of the first six phase-I patients—evidence of target engagement rather than clinical efficacy (133, 134). Recruitment blockade may be particularly valuable when suppressive cells remain abundant systemically: in rhabdomyosarcoma models, genetic or antibody-mediated CXCR2 inhibition prevented trafficking of CXCR2^+^CD11b^+^Ly6G hi MDSCs into tumors and restored the activity of delayed anti-PD-1 treatment, establishing a mechanistic rationale for combining chemokine-axis inhibitors with checkpoint blockade (135). Differentiation-based approaches seek to resolve pathological immaturity rather than eradicate the entire myeloid compartment; accordingly, Dectin-1 ligation by particulate β-glucan induced PMN-MDSC apoptosis while converting monocytic MDSCs into F4/80^+^CD11c^+^ APCs in tumor-bearing mice, whereas a single-arm phase-Ib/II study of intermittent all-trans retinoic acid (ATRA) plus pembrolizumab in 24 anti-PD-1-naïve patients with metastatic melanoma reduced circulating PMN-MDSCs, increased melanoma-reactive T-cell activity and produced a 71% objective response rate, although the uncontrolled, predominantly treatment-naïve population precludes attribution of efficacy specifically to MDSC differentiation (136, 137). Functional reprogramming offers a complementary strategy. For instance, selective FATP2 inhibition with lipofermata interrupted arachidonic-acid uptake and prostaglandin-E_2_ synthesis in PMN-MDSCs and potentiated checkpoint blockade in mice, while the class-I histone-deacetylase inhibitor entinostat suppressed arginase-1, iNOS and COX-2 in both monocytic and polymorphonuclear MDSCs, thereby improving anti-PD-1 efficacy in lung- and renal-carcinoma models (138, 139). More direct depletion has been demonstrated with MDSC-binding peptide–Fc “peptibodies,” which removed circulating, splenic and intratumoral MDSCs across mouse models without depleting dendritic, T, B or NK cells, and with the TRAIL-R2 agonist DS-8273a, which in a 16-patient phase-I study reduced elevated peripheral MDSCs in most participants and intratumoral MDSCs in half of evaluable paired biopsies without measurable loss of mature myeloid or lymphoid populations (140, 141). Targeting suppressive effector pathways may also counteract therapy-induced MDSCs: integrated patient profiling and mouse experiments implicated radiation-induced MDSCs causally in systemic lymphopenia after glioblastoma chemoradiotherapy, with arginase inhibition or the phosphodiesterase-5 inhibitor tadalafil preventing lymphopenia and improving survival in mice; a subsequent phase-Ib study found that tadalafil was tolerable with chemoradiotherapy and was associated with reduced peripheral MDSCs and greater CD8^+^-T-cell proliferation and activation, but did not significantly improve progression-free or overall survival relative to historical controls (30, 142). Finally, MDSC targeting can enhance therapeutic vaccination: in an interim randomized study of 41 patients with extensive-stage small-cell lung cancer, adding ATRA to a wild-type-p53-transduced DC vaccine reduced circulating MDSCs by more than twofold and increased detectable p53-specific responses from 20% with vaccine alone to 41.7%, with granzyme-B^+^ CD8^+^ T-cell expansion confined to the combination arm (143). Collectively, these data support biomarker-guided incorporation of MDSC-directed agents into checkpoint- and vaccine-based regimens.

## 9 Innate and innate-like lymphocytes at the cancer–immunity interface

ILCs, NKT cells and γδ T cells bridge the innate–adaptive interface by combining rapid, germline-encoded responsiveness with cytotoxic and cytokine-mediated functions that shape tissue immunity, yet their effects are exquisitely dependent on anatomical site and tumor stage.

### 9.1 Context-dependent functions of ILC1, ILC2 and ILC3

Innate lymphoid cells (ILCs) are tissue-enriched lymphocytes that lack rearranged antigen receptors and broadly recapitulate type-1, type-2 and type-3 immune programs: T-bet^+^ ILC1s principally produce IFN-γ and TNF, GATA3^+^ ILC2s produce IL-5, IL-9, IL-13 and amphiregulin, and RORγt^+^ ILC3s produce IL-17, IL-22 and lymphotoxin; however, these identities are plastic, making anatomical niche, tumor stage and locally available cytokines stronger predictors of function than subset designation alone. ILC1s can mediate early cancer surveillance, as analyses of human chromophobe renal-cell and breast cancers, together with murine mammary-tumor models, identified a granzyme-A-expressing cytotoxic ILC1 population maintained by cancer-cell-derived IL-15, with IL-15-associated ILC1 signatures correlating with favourable patient survival (144). ILC2 biology is especially context dependent. For instance, in orthotopic pancreatic ductal adenocarcinoma (PDAC), IL-33 activated PD-1^+^ tumor ILC2s, promoted intratumoral DC accumulation and reinforced CD8^+^ T-cell-dependent tumor control and PD-1 blockade, whereas in IL-33-rich colorectal models PPARγ maintained ILC2 mitochondrial fitness and type-2 cytokine production, thereby supporting tumor-cell invasion and growth (145, 146). Moreover, expanded human—but not murine—ILC2s were shown to engage tumor-cell CD112/CD155 through DNAM-1 and release granzyme B, inducing apoptosis or pyroptosis; a scalable expansion platform consequently produced antitumor activity in xenograft models of acute myeloid leukemia, pancreatic and lung cancer, and glioblastoma, although this remains a preclinical cellular-therapy concept (147). ILC3s likewise cannot be reduced to an IL-17-driven protumor phenotype. In colorectal cancer, ILC3-specific MHC-II-dependent dialogue with T cells maintained a microbiota capable of inducing type-1 immunity. By contrast, ILC3 depletion, plasticity or selective MHC-II loss produced invasive disease and resistance to PD-1 blockade in mice. Microbiota from patients with dysregulated ILC3s similarly failed to transfer immunotherapy responsiveness (148). These populations also organize multicellular niches. For instance, a 2025 study demonstrated that IL-33 activates lymphotoxin-expressing ILC2s that migrate from the gut to PDAC, engage LTβR^+^ myeloid organizer cells and initiate tertiary lymphoid structures containing DCs and adaptive lymphocytes; corresponding human ILC2–IL-33 signatures correlated with favourable prognosis, while engineered IL-33 expanded ILC2s and tertiary lymphoid structures and improved tumor control in mice (149). Collectively, epithelial alarmins and tumor-derived cytokines, stromal and myeloid organizer signals, microbial cues and reciprocal interactions with T and B cells determine whether an ILC state sustains immune exclusion or converts tissue inflammation into productive antitumor immunity.

### 9.2 NKT and gammadelta T cells at the innate–adaptive interface

NKT and gammadelta T cells occupy a complementary innate–adaptive interface because they combine germline-biased, rapidly responsive antigen recognition with TCR-dependent specificity: type-1 invariant NKT cells recognize self or microbial glycolipids—including the agonist α-galactosylceramide (α-GalCer)—presented by monomorphic CD1d, whereas the predominant circulating human Vγ9Vδ2 subset senses microbial or transformation-associated phosphoantigens through a composite system in which intracellular BTN3A1 senses phosphoantigen accumulation and BTN2A1 directly binds the germline-encoded Vγ9 TCR region (150, 151). Following activation, both populations can deploy perforin–granzyme and Fas/FasL or TRAIL-mediated cytotoxicity while rapidly releasing IFN-γ, TNF, IL-2 and, particularly from NKT cells, IL-4; these cytokines activate NK cells, license DCs and shape conventional CD8^+^ T-cell responses, thereby allowing direct tumor killing and broader immune orchestration to occur in parallel (152, 153). In a single-arm phase II study of 35 patients with previously treated NSCLC, intravenous α-GalCer-pulsed APCs were well tolerated. The treatment increased NK cells, α-GalCer-responsive IFN-γ-producing cells and effector CD8^+^ T cells. However, it produced only one partial response and did not establish efficacy despite a reported median overall survival of 21.9 months (151). Bispecific engagement offers greater tumor selectivity: a CD1d–Vδ2 engager displayed functionally trispecific activity by stabilizing CD1d–NKT-TCR interactions while recruiting Vγ9Vδ2 cells to CD1d^+^ myeloma, monocytic AML and chronic lymphocytic leukemia cells, inducing cytokine release, expansion and cytolysis in patient-derived samples and improving survival in mouse models; tolerability was examined only with a surrogate engager in non-human primates, so these findings remain preclinical (152). Adoptive platforms have nevertheless generated early clinical signals. For instance, autologous GD2-CAR–IL-15 NKT cells produced no dose-limiting toxicities and achieved a 25% objective response rate—one complete and two partial responses—among 12 children with relapsed or refractory neuroblastoma, whereas 414 infusions of healthy-donor Vγ9Vδ2 cells in 132 patients supported allogeneic feasibility and safety, although the survival analysis was restricted to 18 non-randomized, highly selected recipients and remains hypothesis-generating (154, 155). Most recently, intra-arterial administration of allogeneic induced-pluripotent-stem-cell-derived iNKT cells to ten patients with recurrent head-and-neck cancer caused one dose-limiting grade-3 rash but no other severe treatment-related events; tumor-growth suppression in two patients accompanied by clonal expansion of memory/effector CD8^+^ T cells provides mechanistic evidence of innate–adaptive recruitment, but not yet proof of clinical efficacy (153). Thus, agonist-loaded APCs, innate-like T-cell engagers and engineered or allogeneic cellular products are mechanistically compelling, but randomized efficacy data, reliable intratumoral trafficking and persistence, and control of exhaustion and cytokine polarization remain essential translational requirements.

### 9.3 Mast cells, eosinophils and tissue-resident innate populations

Beyond the canonical macrophage, DC, neutrophil and NK-cell axes, mast cells, eosinophils and anatomically specialized resident phagocytes constitute a numerically modest but functionally consequential layer of tumor immunity whose effects are determined by cellular state, spatial niche, disease stage and treatment exposure rather than abundance alone. In gastric cancer, epithelial IL-33 activates tumor-adjacent mast cells to produce CSF2, CCL3 and IL-6, thereby recruiting macrophages and sustaining proliferation and angiogenesis; under VEGF-directed therapy, mast-cell-derived granzyme B can additionally liberate matrix-bound FGF1 and GM-CSF, while FGF2 secretion restores endothelial proliferation and promotes adaptive angiogenic resistance (156, 157). Mast-cell programs are nevertheless spatially and transcriptionally divergent: mast-cell–Treg-cell-rich melanoma regions were associated with reduced HLA class I expression and exclusion of CD8 + granzyme-B + cells after PD-1 blockade, whereas single-cell and functional analyses of lung adenocarcinoma identified an IL-18 + mast-cell state in which NLRP3–caspase-1–GSDMD signaling enhanced MAIT-cell NKG2D, IFN-γ production and antitumor cytotoxicity (158, 159). Most notably, reverse-translational analyses of triple-negative breast cancer localized antigen-presenting mast cells to tertiary lymphoid structures and demonstrated their capacity for antigen presentation, cross-presentation and costimulation; a subsequent open-label phase II proof-of-concept arm in anti-PD-1-refractory metastatic disease met its prespecified endpoint, with cromolyn plus an anti-PD-1 backbone producing a confirmed objective response rate of 50.0%, although controlled validation is required before this result can be generalized (160). Eosinophils likewise operate as vascular and chemotactic amplifiers: activated eosinophils promoted macrophage repolarization, vascular normalization and tumor-specific CD8 + T-cell recruitment in mouse models, whereas DPP4 inhibition increased CCL11-dependent eosinophil trafficking and revealed tumor-cell IL-33- and eosinophil-degranulation-dependent control of hepatocellular and breast tumors (161, 162). This activity is highly context dependent, because inflammatory eosinophilia promoted experimental pulmonary, bone and intestinal metastasis through eosinophil-derived CCL6–tumor-cell CCR1 signaling, whereas responding patients with metastatic breast cancer—and corresponding mouse models—displayed an ICB-induced CD4 + T-cell–IL-5 axis that expanded eosinophils systemically and an IL-33-dependent program that recruited them intratumorally to support CD8 + T-cell activation (163, 164). Organ-resident macrophages further illustrate how tissue identity governs cancer outcome. For instance, embryo-derived alveolar macrophages maintained disseminated breast cancer cells in dormancy through sustained TGF-β2–TGF-βRIII signaling, whereas microglial RELA–NF-κB activation promoted melanoma brain metastasis and its genetic inhibition reprogrammed microglia toward inflammatory, lymphocyte-recruiting states that improved checkpoint-blockade responses in preclinical models, with supportive correlations in patient specimens (165, 166). Finally, attenuated *Escherichia coli* delivering CRISPR–CasΦ components to Kupffer cells enabled *MafB/c-Maf* disruption, resident-cell expansion, entry into established liver metastases, cancer-cell phagocytosis and activation of antitumor T cells, providing preclinical proof that tissue-resident innate populations can be re-engineered *in situ* rather than merely depleted (167).

Taken together, these findings establish that the therapeutic significance of innate and innate-like immune cells cannot be inferred from lineage identity alone, because macrophages, NK cells, neutrophils, MDSCs, ILCs, innate-like T cells and tissue-resident populations acquire divergent functions according to their developmental origin, activation state, anatomical niche and treatment exposure. **Table 1** consolidates these context-dependent activities, principal therapeutic approaches and current limitations across the major innate immune compartments.

**Table 1:** Context-dependent functions and therapeutic targeting of innate and innate-like immune cells in cancer.

Immune population	Context-dependent roles in cancer	Principal therapeutic approaches	Translational status and limitations	Representative references
DCs	cDC1s cross-present tumor antigens, cDC2s support CD4^+^ immunity and pDCs produce type I interferons; tumor-derived metabolites and cytokines can instead impair antigen processing, maturation and migration.	DC vaccines, FLT3L, receptor-targeted antigen delivery, and CD40 or TLR agonists.	Vaccination can induce robust antigen-specific immunity without improving survival, emphasizing the need to overcome downstream suppression.	(33, 46, 49, 50)
Tumor-associated macrophages	Protective states support phagocytosis and lymphocyte recruitment, whereas SPP1^+^, TREM2^+^ and metabolically adapted states promote immune exclusion, fibrosis, angiogenesis and metastasis.	CSF1R or CCR2 inhibition; CD47–SIRPα, CLEVER-1 or TREM2 blockade; CD40 activation, nanoparticles and engineered macrophages.	Early clinical activity is heterogeneous; nonspecific depletion may remove protective resident populations.	(58, 66, 67, 71)
NK cells	NK cells recognize missing-self and stress ligands, mediate cytotoxicity and antibody-dependent cellular cytotoxicity, and recruit cDC1s; TGF-β, hypoxia, adenosine and ligand loss drive dysfunction.	IL-15 agonists, checkpoint inhibitors, antibodies, multispecific engagers, adoptive NK cells and CAR-NK platforms.	Clinical responses are strongest in hematological malignancies; solid tumors remain limited by trafficking, persistence and metabolic suppression.	(15, 18, 82, 94)
Neutrophils	Interferon-responsive states can support tumor killing, whereas suppressive neutrophils and NETs promote immune exclusion, angiogenesis, metastatic seeding and dormant-cell awakening.	CXCR1/2 or PAD4 inhibition, targeted NET degradation, functional reprogramming and neutrophil-mediated drug delivery.	CXCR1/2 blockade has shown limited clinical efficacy; systemic targeting risks neutropenia and impaired antimicrobial defense.	(100, 104, 112, 114)
MDSCs	PMN- and monocytic MDSCs suppress T-, NK- and DC-cell functions through ARG1, iNOS, ROS, peroxynitrite, adenosine and nutrient restriction.	Inhibition of development or recruitment, differentiation with ATRA, metabolic reprogramming and selective depletion.	Pharmacodynamic effects have been demonstrated, but clinical benefit remains unconfirmed and phenotypic overlap complicates patient selection.	(124, 137, 141, 143)
Innate lymphoid cells	ILC1s can mediate cytotoxic surveillance, whereas ILC2 and ILC3 states may support DC–T-cell immunity and tertiary lymphoid structures or drive type-2/17 inflammation and tumor growth.	Cytokine-guided activation, ex vivo expansion and manipulation of epithelial–stromal–ILC signaling.	Predominantly preclinical; extensive tissue-dependent plasticity limits universal targeting strategies.	(144–149)
NKT and gammadelta T cells	Rapid recognition of CD1d-presented lipids or BTN-associated phosphoantigens enables cytotoxicity and cytokine-mediated activation of DCs, NK cells and conventional T cells.	α-GalCer-loaded APCs, bispecific engagers, allogeneic gammadelta T cells, CAR-NKT cells and iPSC-derived products.	Early studies support feasibility, but efficacy is constrained by trafficking, persistence and limited randomized evidence.	(150–155)
Other tissue-associated innate populations	Mast cells, eosinophils and resident phagocytes can promote antigen presentation, vascular normalization and lymphocyte recruitment or, alternatively, angiogenesis, immune exclusion and organ-specific metastasis.	Mast-cell modulation, eosinophil recruitment and *in situ* reprogramming of microglia, Kupffer cells or other resident phagocytes.	Functions are highly state- and organ-dependent; therapeutic approaches remain early clinical or preclinical.	(156–167)

cDC1/2, conventional type 1/2 dendritic cell; ILC, innate lymphoid cell; MDSC, myeloid-derived suppressor cell; NET, neutrophil extracellular trap; NK, natural killer; pDC, plasmacytoid dendritic cell; PMN-MDSC, polymorphonuclear MDSC.

## 10 Innate immune-sensing pathways: contextual biology and therapeutic targeting

### 10.1 TLRs signaling in cancer

TLRs impose compartment-specific surveillance. Specifically, plasma-membrane TLR1/2/4/5/6 primarily sample extracellular microbial lipids and proteins together with selected damage-associated molecules, whereas endosomal TLR3, TLR7/8 and TLR9 recognize double-stranded RNA, single-stranded RNA and CpG-rich DNA, respectively. Signal propagation through MyD88 and/or TRIF activates NF-κB, AP-1 and interferon-regulatory factors, thereby coupling inflammatory cytokine production to DC maturation, antigen presentation and lymphocyte recruitment; however, response quality depends on ligand chemistry, cellular distribution and whether signaling is acute or chronic. Clinically, a 23-patient randomized phase II study in malignant glioma showed that adding the TLR3/MDA5-active viral mimic poly-ICLC or the TLR7/8 agonist resiquimod to autologous tumor-lysate-pulsed DC vaccination was tolerable and induced systemic interferon and immune-cell activation programs, although the trial was powered for immunological—not survival—end points (168). Likewise, intratumoral delivery of the virus-like-particle-formulated TLR9 agonist vidutolimod with pembrolizumab produced durable responses in 25% of 44 patients with anti-PD-1-refractory melanoma and regression of both injected and non-injected lesions, supporting localized TLR activation as a means of initiating systemic immunity (35). Nevertheless, the 481-patient phase III ILLUMINATE-301 trial found no improvement in objective response or overall survival when intratumoral tilsotolimod was added to ipilimumab, while experimental lung adenocarcinoma demonstrated that malignant-cell—not stromal or immune-cell—TLR7 could recruit MDSCs and accelerate growth and metastasis (169, 170). These findings argue that TLR therapeutics require spatially restricted exposure, pharmacodynamic confirmation in the intended antigen-presenting-cell compartment and careful control of systemic cytokinaemia and tumor-intrinsic protumor signaling.

### 10.2 cGAS–STING signaling in tumor immunity and immune evasion

Cytosolic double-stranded DNA activates cyclic GMP–AMP synthase (cGAS), which produces 2′3′-cGAMP; cGAMP-bound STING subsequently traffics from the endoplasmic reticulum and engages TBK1–IRF3 and NF-κB to induce type I interferons and inflammatory mediators, while transfer of tumor DNA into host APCs provides a mechanistic bridge to DC activation and spontaneous CD8^+^ T-cell priming (171, 172). Recent drug-development strategies address the poor permeability and pharmacokinetics of cyclic dinucleotides. Specifically, the orally active non-nucleotide agonist MSA-2 exhibited enhanced potency under acidic tumor conditions, durable STING-dependent regression and anti-PD-1 synergy in mouse models, whereas intravenously administered tumor-penetrating PEG–lipid nanodiscs improved cyclic-dinucleotide deposition, coordinated antigen and agonist delivery to APCs, and generated systemic immunity and memory (173, 174). Translation has been less compelling: in a 106-patient phase Ib basket study, intratumoral MIW815 plus spartalizumab produced measurable on-target pharmacodynamic activity and a 10.4% objective-response rate but only limited overall antitumor activity (73). This discrepancy reflects profound cellular and temporal context dependence. Chronic tumor-cell-intrinsic activation driven by chromosomal instability can undergo interferon-selective tachyphylaxis and rewire STING output toward ER stress, immunosuppression and metastasis. ENPP1 expressed by malignant and host tissues hydrolyses extracellular cGAMP and restricts beneficial paracrine STING activation (27, 175). Consequently, STING agonism should be developed as an acute, compartment-selective intervention—potentially combined with checkpoint blockade, radiotherapy or ENPP1 inhibition—rather than as indiscriminate pathway activation; chromosomal instability, STING competence, cGAMP metabolism and cell-resolved interferon responses are plausible stratification biomarkers.

### 10.3 RIG-I-like receptor–MAVS signaling

RIG-I preferentially recognizes short duplex RNAs bearing 5′-di- or triphosphate termini, whereas MDA5 assembles along longer duplex RNA; their caspase-activation-and-recruitment domains engage mitochondrial antiviral-signaling protein (MAVS), initiating TBK1–IKKϵ–IRF3/7 and IKK–NF-κB signaling, type I/III interferon production, inflammatory chemokine release and antiviral-like death of susceptible malignant cells. In mouse tumors, tumor-cell-intrinsic RIG-I–caspase-3 activity supported antigen cross-presentation by CD103^+^ DCs, expansion of tumor-specific CD8^+^ T cells and responsiveness to CTLA-4 blockade, while therapeutic 5′-triphosphorylated RNA augmented checkpoint inhibition (176). The structurally defined stem-loop agonist SLR14 was taken up predominantly by intratumoral CD11b^+^ cells, induced CD8^+^ T-cell and NK-cell accumulation, enhanced anti-PD-1 efficacy and controlled injected, contralateral and metastatic tumors with immune-memory formation in mice (177). To overcome nuclease degradation and inefficient cytosolic access, ionizable lipid nanoparticles carrying 5′-triphosphate stem-loop RNA enabled both intratumoral and intravenous administration, suppressed poorly immunogenic melanoma and breast tumors, enhanced anti-PD-1 responses and reduced pulmonary metastatic burden in preclinical models (178). Clinical results remain instructively modest: intratumoral MK-4621 induced interferon-pathway biomarkers but produced no objective responses among 15 monotherapy recipients and three partial responses among 30 patients receiving pembrolizumab, whereas the dual TLR7/8–RIG-I agonist CV8102 produced responses in 5 of 30 anti-PD-1-refractory melanoma patients in its combination-expansion cohort but none of 10 receiving monotherapy—an effect that cannot be attributed specifically to RIG-I because of the agent’s dual-receptor activity (179, 180). Future success will therefore depend on RNA structural specificity, protected cytosolic delivery, simultaneous engagement of malignant and myeloid compartments, and dosing schedules that preserve antiviral-like immunogenicity without provoking uncontrolled systemic inflammation.

### 10.4 Inflammasome signaling and inflammatory cell death

Inflammasomes translate cytosolic danger sensing into inflammatory cytokine release and lytic cell death. Specifically, NLRP3 responds to convergent disturbances such as potassium efflux, lysosomal rupture and mitochondrial dysfunction, whereas AIM2 detects double-stranded DNA, NAIP–NLRC4 recognizes microbial or ectopically expressed ligands, and NLRP1 and pyrin monitor proteostatic or cytoskeletal perturbations; assembly of these platforms recruits ASC and caspase-1, which processes pro–IL-1β and pro–IL-18 and cleaves gasdermin D, while non-canonical caspase-4/5/11 signaling activates gasdermin D and secondarily engages NLRP3. Pyroptosis can also arise independently of canonical inflammasomes when caspase-3 or cytotoxic-lymphocyte-derived granzyme B cleaves gasdermin E (GSDME), thereby converting otherwise apoptotic tumor-cell death into a membrane-permeabilizing, immunogenic process (181). Accordingly, tumor-cell GSDME promoted macrophage phagocytosis and NK- and CD8^+^-cell-mediated control in murine models, whereas radiotherapy-derived tumor DNA and extracellular vesicles activated macrophage AIM2 and NLRP3, producing IL-1β that enhanced DC cross-priming and radiation efficacy (181, 182). The same circuitry can sustain cancer when activation is chronic or occurs in tumor-promoting compartments: tumor-intrinsic PD-L1–PKR–NLRP3 signaling recruited polymorphonuclear MDSCs and limited PD-1 blockade, while phagocytosed β_2_-microglobulin aggregates caused lysosomal rupture and NLRP3-dependent IL-1β/IL-18 production by myeloma-associated macrophages, promoting tumor growth and osteolysis (183, 184). IL-18 is likewise conditional—its endogenous binding protein, IL-18BP, restrains antitumor activity, and an engineered IL-18 variant resistant to this decoy expanded functional NK and CD8^+^ cells in mice; conversely, the phase III CANOPY-1 trial showed that systemic IL-1β neutralization with canakinumab did not improve progression-free or overall survival when added to first-line pembrolizumab–chemotherapy in advanced NSCLC (185, 186). Collectively, these findings argue that inflammasome therapy should be specified by sensor, producing cell, cytokine, gasdermin repertoire and treatment context: localized induction of tumor-cell pyroptosis or DC-supportive IL-1 signaling may be desirable, whereas persistent tumor- or myeloid-cell NLRP3 activity may require selective inhibition rather than indiscriminate systemic blockade.

### 10.5 Complement system

Complement activation occurs through the classical, lectin and alternative pathways, which converge on C3 cleavage and generate C3a, C5a and opsonins such as C3b. C3a and C5a recruit myeloid cells and promote local inflammation. In some contexts, C3b-mediated opsonization promotes phagocytosis and lysis of antibody-coated tumor cells, thereby supporting antitumor immunity (187, 188).

However, the functional outcome of complement activation is context dependent. Tumor-derived C1q can support tumor growth (189). Tumor cells evade complement-mediated attack by expressing CD46, CD55 and CD59, which restrict complement deposition (190). C5a signaling can also recruit and reprogram suppressive myeloid populations and promote tumor progression (191).

Complement-targeted drugs are clinically established in non-malignant complement-mediated disorders, but their role in cancer remains investigational (192). In solid tumors, global blockade may impair antimicrobial defense and remove antitumor effector functions. Rational integration of complement modulation into cancer immunotherapy will require a clearer understanding of compartment-specific complement biology and the development of strategies that distinguish protumor from antitumor complement activities.

## 11 Innate immune programming of therapeutic cancer vaccines

Therapeutic cancer vaccines depend on the same innate processes that govern spontaneous tumor immunity: malignant-cell sensing, antigen uptake and presentation, effector-cell activation, and relief of tumor-microenvironmental suppression. Their efficacy therefore requires coordinated DC activation and adjuvant signaling together with strategies that overcome myeloid and metabolic barriers.

### 11.1 Immunological requirements for effective cancer vaccines

Effective therapeutic vaccination requires the coordinated delivery of antigen and innate stimulation. Specifically, tumor antigens must reach professional APCs, persist long enough for MHC-I and MHC-II presentation, and induce DC maturation, costimulation, lymph-node migration and the priming of cytotoxic CD8^+^ and helper CD4^+^ T cells capable of generating durable memory (193, 194). Recent mechanistic work showed that mRNA–lipid nanoparticle vaccines recruit redundant cDC1- and cDC2-dependent priming pathways and substantial type-I-interferon-dependent transfer of peptide–MHC-I complexes—”cross-dressing”—rather than relying exclusively on canonical WDFY4-dependent cross-presentation; earlier RNA-lipoplex studies similarly demonstrated that systemic delivery to DC-rich lymphoid tissues can harness antiviral type I interferon as an endogenous adjuvant (193, 194). Innate stimulation must nevertheless be calibrated spatially and temporally, because insufficient activation produces poorly costimulatory or tolerogenic APCs, whereas indiscriminate systemic inflammation may narrow the therapeutic window without improving tumor-directed immunity. Vaccine-expanded lymphocytes must subsequently overcome regulatory T cells, suppressive myeloid populations, metabolic restriction, inhibitory checkpoints and tumor-cell defects in antigen presentation; notably, DC vaccination can induce PD-L1^+^ macrophage niches in lymph nodes and tumors that suppress CD8^+^ T cells, with PD-L1 co-blockade restoring tumor control in experimental models (52). Clinically, this rationale is supported by the randomized phase IIb KEYNOTE-942 study, in which individualized mRNA-4157/V940 plus pembrolizumab prolonged recurrence-free survival compared with pembrolizumab alone after resection of high-risk melanoma, although confirmatory phase III evidence remains essential (195). Accordingly, vaccine evaluation should integrate the magnitude, breadth, polyfunctionality, clonality, persistence and tumor trafficking of vaccine-induced T cells with antigen-loss immunoediting and clinical outcomes, rather than relying exclusively on peripheral immune-response assays.

### 11.2 Antigen selection and neoantigen prioritization

Antigen selection determines the balance between specificity, population coverage, manufacturability and escape. Specifically, tumor-associated and shared antigens support predefined, off-the-shelf products but are constrained by self-tolerance, heterogeneous expression and frequently by HLA restriction, whereas private neoantigens offer greater tumor specificity at the cost of individualized sequencing, prediction and manufacturing (196, 197). The TESLA consortium identified tumor expression, peptide–MHC binding affinity and complex stability as major correlates of neoepitope immunogenicity, while longitudinal analysis of human pancreatic cancers demonstrated selective editing of high-quality neoantigens, emphasizing antigen quality—not mutation number alone—as a determinant of immune pressure (198, 199). Antigen-discovery pipelines should therefore integrate matched tumor–normal exome or genome sequencing, RNA expression, HLA typing and mass-spectrometric immunopeptidomics; NeoDisc illustrates how this strategy can prioritize naturally presented HLA-I and HLA-II antigens, including viral and noncanonical candidates, while detecting HLA loss or other defects in antigen-processing machinery that may invalidate otherwise attractive targets (196). The limitations of shared-antigen vaccination were evident in ATALANTE-1: the HLA-A2-restricted nine-peptide OSE2101 vaccine did not significantly improve overall survival in the full randomized NSCLC population, although a secondary-resistance subgroup experienced longer survival and less severe toxicity than chemotherapy; premature COVID-19-related termination and subgroup analysis require confirmation (197). ELI-002 2P elicited mKRAS-specific responses in 21 of 25 patients; among evaluable patients, 71% generated both CD4+ and CD8+ responses and 67% showed antigen spreading. Associations with relapse-free and overall survival remain hypothesis-generating in this small single-arm phase I trial (200). Rational formulations should thus target several expressed, clonally conserved and preferably driver-associated antigens, include both HLA-I and HLA-II epitopes, and preserve sufficient breadth to limit immunoediting and inter-lesional escape (196, 200).

### 11.3 Vaccine platforms and delivery technologies

Vaccine platforms differ in antigenic breadth, manufacturability, delivery route and reliance on adjuvant support. Synthetic peptide and protein vaccines are chemically defined and scalable but depend strongly on adjuvant selection, antigen persistence and efficient DC uptake; OSE2101 and albumin-hitchhiking ELI-002 exemplify conventional HLA-restricted and lymph-node-targeted peptide designs, respectively (197, 200). Cell-based formulations exchange manufacturing simplicity for antigenic breadth: tumor-lysate-loaded autologous DCVax-L was associated with longer survival than external controls in glioblastoma, but its nonrandomized, externally controlled phase III analysis limits causal interpretation, whereas the GM-CSF-secreting allogeneic whole-cell vaccine GVAX combined with nivolumab and urelumab increased intratumoral activated CD8^+^CD137^+^ cells in resectable pancreatic cancer without establishing a statistically significant survival benefit in the small platform study (201, 202). Personalized uridine-mRNA lipoplexes generated neoantigen-reactive T cells in 8 of 16 patients with resected pancreatic cancer, and subsequent tracking demonstrated cytotoxic, tissue-resident-memory-like CD8^+^ clones persisting for as long as three years; the apparent association with delayed recurrence nevertheless derives from a small, nonrandomized cohort (203, 204). DNA vaccination is stable and readily multiplexed: in advanced HCC, individualized GNOS-PV02 encoding as many as 40 neoantigens, combined with plasmid IL-12 and pembrolizumab, induced vaccine-specific responses in 19 of 22 evaluable patients and produced a 30.6% response rate, although the single-arm design cannot isolate the vaccine contribution (205). Heterologous chimpanzee-adenovirus priming followed by self-amplifying-mRNA boosting likewise generated durable, polyfunctional neoantigen-specific T cells with acceptable tolerability in an interim phase I study, but antitumor efficacy was not established (206). Liposomes, nanoparticles, amphiphiles and other biomaterials can co-deliver antigen and danger signals, direct cargo to lymph nodes or selected APCs, and regulate exposure kinetics, as illustrated by systemic RNA lipoplexes and albumin-binding ELI-002 (194, 200). Finally, *in situ* vaccination can convert the tumor itself into an antigen source: intratumoral FLT3 ligand, localized radiotherapy and the TLR3 agonist poly-ICLC recruited and activated DCs, produced systemic regressions in some patients with indolent lymphoma and enhanced PD-1 blockade preclinically (44). Platform selection should therefore be guided not merely by payload capacity, but by anatomical delivery, innate-sensing profile, manufacturing time, antigenic breadth, repeat-dosing feasibility and the strength of the supporting clinical evidence.

### 11.4 Innate adjuvants and costimulatory programming

Innate adjuvants should be selected as instructive signals that determine antigen-presenting-cell activation, cross-presentation and T-cell differentiation, rather than as interchangeable amplifiers of inflammation. Clinically, a randomized phase II study in 23 patients with malignant glioma, showed that adding poly-ICLC or resiquimod to tumor-lysate-pulsed DC vaccination was tolerable and induced systemic interferon programs; poly-ICLC produced the more coherent response, although the trial was powered for immunological rather than survival endpoints (168). STING agonism can be coupled directly to antigen trafficking: the SABER platform delivered conjugated antigens to the DC endoplasmic reticulum, assembled cross-presentation machinery into membrane “microreactors” and elicited substantially stronger neoantigen-specific CD8^+^ responses than unconjugated antigen–agonist mixtures (207). Cytosolic 5′-triphosphate RNA similarly enhanced DC cross-priming through host RIG-I–MAVS–type-I-interferon signaling and cooperated with CTLA-4 blockade in murine melanoma, whereas a covalently linked TLR7–NOD2 agonist demonstrated that coordinated pattern-recognition-receptor engagement can promote autophagy and antigen-specific cellular immunity, albeit in a non-oncological mucosal-vaccine model (208, 209). Cytokine-encoding adjuvants provide a complementary means of specifying lymphocyte fate: an IL-12 mRNA–LNP module, augmented antigen-specific CD8^+^-cell expansion, effector activity and circulating and tissue-resident memory and improved protection against B16F0-OVA melanoma challenge in mice (210). Costimulatory agonists must likewise be scheduled rather than merely added, as liposomal peptide–STING priming followed one week later by peptide plus agonistic CD40 antibody drove massive T-cell expansion and regression of established MC38 tumors (211). Adjuvant choice should therefore integrate tumor accessibility and innate-sensor competence with antigen format and desired immunity.

### 11.5 Lymphoid-tissue targeting and controlled vaccine delivery

Nanocarrier size, surface charge, composition and ligand display influence drainage, cellular uptake and intracellular trafficking; their effects depend on formulation, administration route and biological context. Carrier specifications should therefore be optimized empirically for the target cell and delivery route.

Mechanism-based nanocarriers can enhance antitumor immunity by coordinating antigen–adjuvant delivery, improving tumor penetration, supporting immune-cell function, and promoting immunogenic cell death, with designs tailored to distinct innate immune populations (212–215). This principle is exemplified by a recently reported metal-coordination-driven nanoparticle that co-delivers the STING agonist SR717 with the chemotherapeutic agent curcumin, improving agonist bioavailability, inducing immunogenic cell death, and reversing immunosuppressive metabolism through IDO1 inhibition (215). The same concept extends to immune cell therapies more broadly, with lipid nanoparticles used for *in vivo* immune cell stimulation and nanoparticle-mediated gene editing and cytokine delivery to enhance cell fitness (214).

Emerging biomimetic strategies address tumor penetration limitations: cell-membrane-coated photosensitizers preserve natural surface properties to improve tissue permeability and antitumor immune responses (213). These approaches can be integrated with regulated cell death pathways—including ferroptosis, pyroptosis and necroptosis—to amplify immunogenic cell death during sonodynamic therapy and overcome apoptosis resistance (212). Collectively, these examples illustrate that nanocarrier development should move beyond passive encapsulation toward active, mechanism-based design that accounts for the distinct biology of cDC1s, macrophages and other innate populations.

Delivery systems must co-localize antigen and danger signals in the same competent APC while protecting nucleic-acid cargo, enabling endosomal escape and directing exposure to tumor-draining lymphoid tissue. Intravenously administered, tumor-penetrating PEG–lipid nanodiscs carrying a cleavable STING agonist achieved broad intratumoral distribution and promoted co-localization of tumor antigen and agonist in DCs, producing Batf3- and CD8^+^-T-cell-dependent tumor rejection at substantially lower agonist doses than soluble comparators (174). Cellular and subcellular specificity can provide additional gains. For example, anti-CLEC9A–NY-ESO-1 antibodies delivered multiple epitopes selectively to human CD141^+^ cDC1s for cross-presentation to naïve and melanoma-patient memory CD8^+^ T cells, whereas SABER showed that completing the final cytosol-to-endoplasmic-reticulum delivery step can outperform tissue-level targeting alone (207, 216). For mRNA vaccines, the LiNx nanofiber–hydrogel matrix retained and gradually released mRNA–LNPs, recruited and transfected APCs and generated, with one injection, immune responses and murine antitumor activity comparable or superior to three administrations of free LNPs (217). These studies illustrate distinct route-dependent solutions: systemic nanodiscs favor disseminated tumor access, whereas subcutaneous biomaterial depots create local antigen-expression and immune-cell-interaction niches (174, 217). Dose and schedule should consequently be optimized against antigen-expression kinetics, draining-node biodistribution and innate pharmacodynamic signatures rather than maximum tolerated dose; critically, the superior activity of delayed CD40 boosting after STING priming demonstrates that treatment order can determine whether costimulation expands an established antigen-specific population or is delivered before adequate priming (211). Route, timing and booster interval should ultimately be treated as platform-specific variables, with intratumoral delivery suited to accessible immune-cold lesions, lymph-node-directed administration to efficient *de novo* priming, and systemic formulations to multifocal disease provided that hepatic sequestration and inflammatory exposure are controlled.

### 11.6 Mechanism-based combination strategies

Combination therapy is most compelling when each component repairs a distinct limiting step—priming, trafficking, effector restraint or microenvironmental suppression—rather than simply stacking inflammatory stimuli. In urothelial cancer, the phase I PGV001 study established the feasibility and safety of personalized peptide vaccination with atezolizumab and detected neoantigen-specific T-cell responses in all treated participants, but its small, non-randomized adjuvant and metastatic cohorts cannot establish clinical efficacy (218). In advanced non-squamous NSCLC, NEO-PV-01 administered with carboplatin, pemetrexed and pembrolizumab was feasible, immunogenic and associated with no treatment-related serious adverse events in a 38-patient phase Ib study, although the absence of a comparator prevented separation of the vaccine contribution from chemoimmunotherapy (219). A 2026 single-arm phase II study combining GVAX, pembrolizumab and stereotactic radiotherapy after induction chemotherapy in locally advanced pancreatic cancer reported a median distant-metastasis-free survival of 9.8 months and a 44.4% resection rate, but did not meet its prespecified 13.6-month primary benchmark and remains vulnerable to selection and resection-associated confounding (220). Targeted agents may also enhance vaccination through host rather than tumor-intrinsic effects: pharmacological PIKfyve inhibition activated non-canonical NF-κB signaling in CD11c^+^ cells, increased antigen presentation and potentiated poly(I:C)-associated tumor control in mice, providing a preclinical rationale for biomarker-selected vaccine–kinase-inhibitor combinations (221). Innate-cell reprogramming is particularly relevant where vaccination expands suppressive myeloid states; in murine neoantigen-vaccine models, TREM2 blockade reduced CX3CR1^+^CD206^+^ macrophages, increased functional neoantigen-specific CD8^+^ cells and improved tumor control (222). Oncolytic adenovirus, Maraba virus, vesicular stomatitis virus and vaccinia virus can meanwhile function as interchangeable prime–boost adjuvants when co-administered with mutant peptides, producing CD8-dependent antitumor effects without requiring a separately engineered virus for every personalized antigen set (223). Finally, the BNT211-01 phase I trial showed that a CLDN6-encoding RNA lipoplex vaccine could be administered with CLDN6 CAR-T cells in heavily pretreated solid tumors with manageable toxicity and transient CAR-T-cell expansion; however, heterogeneous cohorts, variable lymphodepletion and delayed vaccine administration precluded determination of CARVac’s independent efficacy (224). Future development should therefore prioritize biomarker-enriched, randomized or factorial studies that preserve tumor-draining lymphatics and prospectively test treatment sequence, distinguishing genuine mechanistic complementarity from additive toxicity.

Clinical benefit requires more than measurable immunogenicity. Durable tumor control also depends on antigen quality, DC programming, administration route and schedule, and relief of tumor-mediated immunosuppression. **Table 2** summarizes selected late-phase studies of therapeutic cancer vaccines.

**Table 2:** Recent Phase III and IV studies on therapeutic cancer vaccines with clinical reference value.

Sponsor	Drug combination	Target disease	Trial outcomes	Trial ID	Representative references
IO Biotech (pembrolizumab supplied by Merck)	IO102–IO103 (Cylembio) + pembrolizumab vs pembrolizumab	Previously untreated unresectable or metastatic melanoma	PFS HR 0.77 (P = 0.0558), not significant; no added toxicity.	NCT05155254 Phase III (IOB-013/KN-D18)	(225)
Radboud University Medical Center	Autologous natural dendritic-cell vaccine vs saline placebo	Resected stage IIIB/C melanoma	No RFS benefit despite T-cell responses (2-year RFS 36.8% vs 46.9%, P = 0.31).	NCT02993315 Phase III (MIND-DC)	(49)
OSE Immunotherapeutics	OSE2101 (Tedopi) vs docetaxel or pemetrexed	HLA-A2-positive advanced NSCLC after chemotherapy and checkpoint blockade	OS not improved overall; HR 0.59 in secondary-resistance subgroup.	NCT02654587 Phase III (ATALANTE-1)	(197)
Amgen	Talimogene laherparepvec (T-VEC) + pembrolizumab vs placebo + pembrolizumab	Unresectable stage IIIB–IVM1c melanoma	No PFS or OS benefit (PFS HR 0.86; OS HR 0.96).	NCT02263508 Phase III (MASTERKEY-265)	(226)
SillaJen	Pexastimogene devacirepvec (Pexa-Vec) followed by sorafenib vs sorafenib	Previously untreated advanced HCC	Terminated for futility (median OS 12.7 vs 14.0 months).	NCT02562755 Phase III (PHOCUS)	(227)
Northwest Biotherapeutics	DCVax-L + standard radiochemotherapy/temozolomide	Newly diagnosed and recurrent glioblastoma	Longer OS in externally controlled analyses; crossover limits causal inference.	NCT00045968 Phase III	(201)
Bavarian Nordic	PROSTVAC-V/F ± GM-CSF vs placebo	Asymptomatic or minimally symptomatic metastatic castration-resistant prostate cancer	Terminated for futility (median OS ~34 months across arms).	NCT01322490 Phase III (PROSPECT)	(228)
Celldex Therapeutics	Rindopepimut + GM-CSF + temozolomide vs control vaccine + temozolomide	Newly diagnosed EGFRvIII-positive glioblastoma	No survival benefit (median OS 20.1 vs 20.0 months; HR 1.01).	NCT01480479 Phase III (ACT IV)	(229)
OSE Immunotherapeutics	OSE2101 (Tedopi) vs docetaxel	HLA-A2-positive metastatic NSCLC with secondary resistance to checkpoint blockade	Ongoing (n≈363); OS primary endpoint; results pending.	NCT06472245 Phase III (ARTEMIA)	(230)
Dendreon	Sipuleucel-T; subsequent anticancer treatment permitted	Asymptomatic or minimally symptomatic metastatic castration-resistant prostate cancer	Observational registry: median OS 30.7 months; no concurrent control.	NCT01306890 Phase IV (PROCEED)	(231)

T-VEC and Pexa-Vec are formally oncolytic-virus immunotherapies rather than conventional antigen-formulated vaccines. CVE, cardiovascular event; DFS, disease-free survival; GM-CSF, granulocyte–macrophage colony-stimulating factor; HCC, hepatocellular carcinoma; HR, hazard ratio; mCRPC, metastatic castration-resistant prostate cancer; NSCLC, non-small-cell lung cancer; OS, overall survival; PFS, progression-free survival; RFS, recurrence-free survival; TMZ, temozolomide.

## 12 Conclusion

Collectively, the evidence reviewed here establishes innate immunity as a dynamic, tissue-embedded control system whose effects cannot be inferred from cell lineage or pathway activation alone. Specifically, depending on anatomical compartment, metabolic state, signal strength and disease stage, the same innate population may eliminate transformed cells, sustain adaptive priming or reinforce tolerance, fibrosis and metastatic fitness. Spatial restriction and mechanistic sequencing will be equally important. The next phase of development should therefore replace indiscriminate innate stimulation with precision orchestration—combining cell-resolved and spatial biomarkers, tumor-restricted or lineage-selective delivery, schedules aligned with antigen release and DC priming, and randomized trials incorporating mechanistic pharmacodynamic endpoints while preserving antimicrobial and tissue-homeostatic functions. Ultimately, innate immunity should be viewed not as an adjunct to T-cell-directed therapy or vaccination, but as the connective framework that determines whether tumor recognition, antigen presentation, effector recruitment and immunological memory culminate in durable clinical control.
